# Fingerprinting of Proteins that Mediate Quagga Mussel Adhesion using a *De Novo* Assembled Foot Transcriptome

**DOI:** 10.1038/s41598-019-41976-7

**Published:** 2019-04-19

**Authors:** David J. Rees, Arash Hanifi, Angelico Obille, Robert Alexander, Eli D. Sone

**Affiliations:** 1 Institute of Biomaterials & Biomedical Engineering, University of Toronto, Toronto, ON Canada; 2 Department of Materials Science & Engineering, University of Toronto, Toronto, ON Canada; 3 Faculty of Dentistry, University of Toronto, Toronto, ON Canada

**Keywords:** Proteomics, Invasive species

## Abstract

The European freshwater mollusk *Dreissena bugensis* (quagga mussel), an invasive species to North America, adheres to surfaces underwater via the byssus: a non-living protein ‘anchor’. In spite of its importance as a biofouling species, the sequence of the majority of byssal proteins responsible for adhesion are not known, and little genomic data is available. To determine protein sequence information, we utilized next-generation RNA sequencing and *de novo* assembly to construct a cDNA library of the quagga mussel foot transcriptome, which contains over 200,000 transcripts. Quagga mussel byssal proteins were extracted from freshly induced secretions and analyzed using LC-MS/MS; peptide spectra were matched to the transcriptome to fingerprint the entire protein primary sequences. We present the full sequences of fourteen novel quagga mussel byssal proteins, named *Dreissena bugensis* foot proteins 4 to 17 (Dbfp4–Dbfp17), and new sequence data for two previously observed byssal proteins Dbfp1 and Dbfp2. Theoretical masses of the newly discovered proteins range from 4.3 kDa to 21.6 kDa. These protein sequences are unique but contain features similar to glue proteins from other species, including a high degree of polymorphism, proteins with repeated peptide motifs, disordered protein structure, and block structures.

## Introduction

The European freshwater mollusk *Dreissena bugensis* (quagga mussel), a close relative of *Dreissena polymorpha* (zebra mussel), was introduced to the Great Lakes in the 1980s and has since rapidly spread throughout eastern North America^1–3^. Both species, like marine mussels, adhere to a plethora of surfaces of differing chemical properties via the byssus: a non-living proteinacious ‘anchor’^4,5^. These mussels have already had a profound economic and ecological impact due to their ability to carpet virtually all underwater surfaces^6,7^. There is thus a pressing need to understand mussel adhesion in order to create targeted anti-fouling coatings, yet very little is known about the quagga mussel byssal proteins that mediate adhesion.

In contrast with freshwater mussels, the marine mussel adhesion system has been extensively studied^8–10^. Marine adhesion has in large part been attributed to the unusual amino acid, 3,4-dihydroxyphenylalanine (DOPA). DOPA has been demonstrated to play important roles in adhesion and cohesion in the marine mussel byssus^9^. Mefp3 and Mefp5, two adhesive proteins in the marine mussel *Mytilus edulis*, contain ~20 and ~28 mol% DOPA, respectively^11,12^. Although the byssi of marine and freshwater mussels are physically similar, containing multiple threads terminating in adhesive plaques, there are significant compositional differences. Freshwater mussel byssi contain low levels of DOPA (<1 mol%)^13^. The evolutionary divergence between marine and freshwater mussels and the recent discovery of unique zebra mussel byssal proteins further suggest there may be differences in the molecular mechanisms of adhesion^14,15^.

Zebra mussels first colonized the Great Lakes; however, quagga mussels are now the dominant *Dreisenna* species in all the Great Lakes except for Lake Superior^16–20^. Four *Dreissena bugensis* foot proteins (Dbfp) have been identified using SDS-PAGE and DOPA-specific NBT staining by Waite and co-workers: Dbfp0 (>200 kDa), Dbfp1 (~69 and ~80 kDa), Dbfp2 (~30 kDa), Dbfp3 (12–13 kDa)^13,21^. Dbfp1-Dbfp3 appear to be homologs of zebra mussel proteins (Dpfp1-Dpfp3), which stain at similar weights to Dpfp1 – Dpfp3. Dbfp1 has been determined to contain 0.6 mol% of DOPA (with a maximum of 2.8 mol% in some fractions) and has been partially sequenced, containing the repetitive motif: DKYPGGGN^13,21^. The amino acid composition of Dbfp2 has been determined, however it has not been sequenced^13^.

In our previous work, quagga mussels were induced to secrete fresh byssal material, which has reduced crosslinking, enabling increased protein extraction^15^. Using this method, we identified several proteins using Tris-Bis SDS-PAGE, including multiple novel proteins. To localize byssal proteins, MALDI-TOF mass spectrometry was used to identify plaque-specific proteins, and interface-specific proteins. A range of proteins was observed from 4.3–18.0 kDa. However, MALDI-TOF does not provide sequence-level information.

In order to determine the primary sequence of byssal proteins, we paired massively parallel RNA sequencing technologies with high-resolution liquid chromatography tandem mass spectrometry (LC-MS/MS)^22^. This type of transcriptomic and proteomic analysis has emerged as a powerful method of analyzing the protein component of biological glues^22–36^. We first created a transcriptome library from the quagga mussel foot, the organ that secretes the byssal proteins. Using LC-MS/MS, extracted byssal proteins fragments were analyzed, and the resulting spectra were matched to the foot transcriptome library to fingerprint the protein primary structure for analysis.

## Methods

### Quagga mussel foot RNA extraction

Quagga mussels (QM) were collected from the mouth of the St. Lawrence River at Kingston, Ontario. Mussels were kept in circulating artificial freshwater at ~12 °C for up to four months and fed powdered green algae^37^. Three quagga mussel feet were harvested to ensure coverage of allelic variation in the local population. Following dissection on ice, each foot was rinsed thoroughly with MilliQ and RNase-Free water, and then submerged in 1 mL of ice-cold TRIzol Reagent (Invitrogen). Samples were homogenized using the BioSpec Mini-Beadbeater-16 with three 2.3 mm chrome-steel beads via 30-second cycles performed 6 times, followed by a 3–5 minute incubation at room temperature. To remove insoluble tissue components, samples were centrifuged for 10 minutes at 12,000 g at 4 °C. The remaining RNA isolation steps were performed as described by Chemozynski and Sacchi^38^, with an additional final 75% ethanol washing steps. RNA was air-dried and resuspended in RNase/DNase-Free water. RNA purity and concentration were determined according to UV absorbance at 260 nm and 280 nm (Nanodrop ND-1000 Spectrophotometer, Thermo Scientific). RNA was then immediately flash-frozen and stored in liquid N_2_ until further use.

### Illumina RNA-sequencing and transcriptome library construction

Next-generation sequencing was completed at The Centre for Applied Genomics (TCAG) at the Hospital for Sick Children in Toronto, Ontario. RNA quality was assessed on a Bioanalyzer 2100 RNA Nano Chip following the manufacturer’s recommendation (Agilent Technologies). Library preparation was performed with the Illumina TruSeq RNA Sample Preparation V2 Guide (Rev. D, September 2012), following the recommended protocol. Libraries were checked on a Bioanalyzer 2100 DNA High Sensitivity Chip (Agilent Technologies) to check for size and quantified by qPCR using the Kapa Library Quantification Illumina/ABI Prism Kit (KAPA Biosystems) following the manufacturer’s recommended protocol. Libraries were pooled in equimolar quantities and paired end sequenced on an Illumina 2500 platform using a Rapid Run Mode Flow Cell and the V3 sequencing chemistry following Illumina’s recommended protocol to generate paired-end reads of 150-bases in length (150 × 2). FASTQ outputs from all three samples were combined for assembly. For each sample, raw reads were preprocessed for adapter/quality trimming and size selection using trim_galore version 0.2.8^39^. Adapter trimming was performed with stringency 5. In addition, trim-galore was used to trim low-quality ends from reads with a minimum Phred score cut-off of 20. Preprocessed reads were pooled together and assembled using Trinity^40^. Mind Trinity created ‘components’ where similar contigs can cluster to, roughly corresponding to ‘genes’ (transcription loci), and the clustered contigs correspond to different transcripts/isoforms of the ‘gene’, referred to as variants in this paper. The FASTA transcriptome library was virtually translated in all six reading frames using in-house scripts. RSEM package was used to perform abundance estimation for assembled transcripts^41^. Initially, full reads were aligned in paired-end mode. In order to accommodate smaller contigs of length < 200 base pairs (bp), each left read was divided into 50 bp sequences and aligned using single-end mode. Due to the zebra mussel and quagga mussel both being part of the *Dreissenidae* family, each assembled contig was aligned against Dreissena_polymorpha mRNA library created by Xu and Faisal using BLAST to identify homologous proteins^42^.

### Protein extraction via induced byssus thread/plaque secretion

To induce the mussel to secrete protein, the foot was injected with ~0.3 mL KCl as previously described^43,44^. After five minutes, the induced thread and plaque (TP) were pulled from the ventral groove, washed in a drop of deionized water, placed on the tip of a glass pestle, and homogenized for two minutes in a 1 mL ground glass hand-held tissue grinder containing either 1% acetic acid or extraction buffer kept on ice. For whole TP analysis containing both insoluble and soluble byssal components, secreted protein was homogenized in 1% acetic acid to prevent oxidation, probe sonicated, flash-frozen in liquid nitrogen, and then stored at −20 °C until trypsin digestion (described below). To extract soluble proteins, secreted proteins were homogenized in basic extraction buffer (EB) was adapted from Rzepecki and Waite^13^, containing 0.2 M sodium borate, 4 M urea, 1 mM KCN, 1 mM EDTA, and 10 mM ascorbic acid. Following extractions from 10–15 mussels, the homogenate was rinsed with 50–100 μL of fresh EB, and pooled. The homogenate was sonicated on ice 20 times with two-second intervals with a probe sonicator, then centrifuged at 17,000 g for eight minutes at 4 °C. Soluble proteins were removed as the supernatant, flash-frozen in liquid nitrogen and stored at −20 °C until separation by SDS-PAGE as described below.

### Bis-tris sodium dodecyl sulfate polyacrylamide gel electrophoresis

The soluble byssal extract from two extraction rounds (20 TP total) was desalted and concentrated via three rounds of ultra-centrifugal filtration using 10% acetonitrile in water with Amicon filters with a cutoff particle size of 3 kDa (EMD Millipore, Billerica, MA, USA). Gel electrophoresis was performed using Life Technologies Bolt 12% Bis-Tris pre-cast 10 well gels, Bolt MES-SDS running buffer (Bis-Tris SDS-PAGE), and Bolt LDS sample buffer. Gels were run in a Bolt Mini Gel Tank, with a constant 165 V voltage setting, for 35–40 minutes and stained with SimplyBlue Safestain (Invitrogen). High-intensity stained gel bands at ~6, ~7, ~14, and ~28 kDa were clipped, de-stained, and stored in 1% acetic acid at 4 °C until use.

### Trypsin digestion whole thread plaques and gel bands for LC-MS/MS

Gel band digestion and whole TP protein digestion and LC-MS/MS were completed at the SickKids SPARC BioCentre at the Hospital for Sick Children. For whole TP, the homogenate was evaporated to dryness, resuspended in 100 mM Tris buffer with 6 M urea, reduced with 200 mM DTT (60 min, RT), and alkylated with 200 mM iodoacetamide (60 min, RT). Urea concentration was diluted to 0.6 M, and then digested with 20 µg trypsin overnight at 37 °C. The reaction was stopped by adjusting the pH < 6. Extracted peptides were dried and reconstituted in 20 µL 0.1% formic acid in water for LC-MS/MS. Gel bands were washed with 50 mM ammonium bicarbonate followed by shrinking with 40% acetonitrile/25 mM ammonium bicarbonate. Samples were reduced with 10 mM DTT (30 min, 56 °C) and alkylated with 100 mM iodoacetamide (15 min, dark, RT) followed by shrinking with 50% acetonitrile/25 mM ammonium bicarbonate (15 min). Samples digested with 13 ng/μL trypsin (Porcine, Sequencing Grade, Promega) overnight at 37 °C and the liquid was collected. Peptides were extracted by vortexing sample separately with 25 mM ammonium bicarbonate, 5% formic acid, 100% acetonitrile, 5% formic acid and 100% acetonitrile and all supernatants were pooled together. Extracted peptides were lyophilized by SpeedVac centrifugation and resuspended in 20 μL 0.1% formic acid in water for LC-MS/MS analysis.

### Liquid chromatography tandem mass spectrometry (LC-MS/MS)

Digested peptides were loaded onto a 100 μm ID pre-column (Dionex) at 4 μL/min and separated over a 50 μm ID analytical column (C18 2 μm, Dionex). The peptides were eluted over 60 min at 250 nL/min using a 0 to 35% acetonitrile gradient in 0.1% formic acid using an EASY n-LC 1000 Nano-Chromatography pump (Thermo Fisher, Odense Denmark). The peptides were then eluted into an electrospray ionization Orbitrap Q-Exactive mass spectrometer (Thermo-Fisher, Bremen, Germany) operated in a data-dependent mode. Data were acquired at 70,000 FWHM resolution in the MS mode and 17,500 FWHM in the MS/MS mode. A total of 10 MS/MS scans were obtained per MS cycle.

### Protein identification, selection, and analysis

Using the proteomics software PEAKS7 (Bioinformatics Solutions Inc., Waterloo, Ontario, Canada), *de novo* sequences derived from MS/MS data were matched against the quagga mussel foot transcriptome with tyrosine hydroxylation to DOPA set as a variable modification. Parent ion and fragment ion mass tolerances were set to 5 ppm and 0.01 Da respectively and hits were manually confirmed by inspecting the spectra. In PEAKS, the identification probabilities of the protein and peptide matches are indicated by the formula −10LogP. Our acceptance criteria for significant matches were a peptide -LogP ≥ 15, protein −10LogP ≥ 50, and *de novo* average local confidence (ALC) score ≥ 80%, with at least 2 peptide spectra identification, unless otherwise stated. Signal peptides were searched using SignalP 4.1^45^. Proteins were examined for homology using NCBI Protein BLAST using no adjustment method to compensate for amino acid composition and filtering low complexity regions^46^. Conserved domains were predicted using SMART (Simple Modular Architecture Research Tool)^47^. The transcriptome library was also searched using the component numbers fingerprinted by LC-MS/MS to discover additional potential protein variant sequences that may have not been expressed or observed in the samples. Variants were aligned using Clustal Omega online tool from the European Bioinformatics Institute^48^. The theoretical mass, pI and amino acid composition of virtual EST protein matches were determined using EMBOSS Pepstats^49^, and Kyte-Doolittle Hydropathy Plots were obtained using ExPASy ProtScale^50^. Repeated sequences and motifs were identified manually and by using SAPS (Statistical Analysis of Protein Sequences)^51^. Protein disorder was analyzed using IUPred, a predictor for intrinsically disordered proteins^52^.

## Results and Discussion

### Quagga mussel foot transcriptome library construction and LC-MS/MS byssal protein identification

In order to determine the primary sequence of quagga mussel byssal proteins, a bottom-up proteomics approach utilizing next-generation sequencing paired with LC-MS/MS protein identification was used. mRNA sequence data from three quagga mussel feet were pooled for *de novo* assembly using Trinity. A minimum 90% of reads from each sample were successfully mapped to the assembled library, indicating a high-quality assembly (Supplemental Table S1). Mind Trinity assembled the long continuous regions of DNA (contigs) into 122,606 components (analogous to genes), containing 207,239 transcripts, analogous to protein variants or isoforms. The assembled library and sequence reads were deposited into the DNA Data Bank of Japan (DDBJ). For all transcripts, the median contig length was 386 bp, and the N30 and N50 contig lengths are 2,396 bp and 1,481 bp, respectively (Supplemental Fig. S1). BLAST analysis of the Xu and Faisal zebra mussel foot cDNA library^42^ against the new quagga mussel transcriptome library revealed that > 90% of the zebra mussel foot transcripts display homology to the quagga mussel foot transcripts. The cDNA library was translated in all six reading frames to create the quagga mussel transcriptome protein library utilized to fingerprinting proteins sequences using LC-MS/MS, described below.

Homogenized freshly-secreted byssal material as well as gel bands from extracted soluble proteins (~6, ~7, ~14, and ~28 kDa) (Supplemental Fig. S2) were digested by trypsin. The resulting peptide fragments were analyzed by LC-MS/MS and matched to the quagga mussel transcriptome library to fingerprint the protein sequences (Table 1). Over 1000 proteins were identified in the whole TP extract. Approximately 200 proteins were identified in each of the ~6, ~7, and ~14 kDa gel bands. There were no confident protein matches in the ~28 kDa band, which could be a result of problems extracting sufficient protein from the gel.

**Table 1:** Summary of LC-MS/MS spectra data and protein matches to the quagga mussel transcriptome library.

Sample	MSScans	MS/MS Scans	Spectrum matches	*De novo* only spectra	Protein Groups	Proteins
Whole TP	17,999	15,282	2,958	1,414	599	1,066
6 kDa	10,272	5,909	201	129	85	202
7 kDa	9,603	7,249	299	239	117	241
14 kDa	9,975	6,510	157	283	107	192

Several criteria were used to identify novel byssal proteins (Table 2) from this large list. Known cellular protein contaminants were removed following identification by BLAST analysis. Proteins that produced BLAST results to known proteins with an expect value below e^−6^ were not considered as novel byssal proteins. In addition, only proteins including a signal peptide, indicative of a secreted protein, were included in the final list. These criteria were chosen to select for the most likely novel byssal proteins but may falsely eliminate some matches. As such, proteins that had a high number of spectral matches but did not meet our acceptance criteria for novel byssal proteins are included in Supplemental Table S2.

**Table 2:** Byssal proteins identified by LC-MS/MS analysis using the foot transcriptome library.

Protein name	Protein Sequence (# of amino acids)^a,b^ Protein variants molecular weight	MW, pI^a^	Score^c^ (−10LogP)	Spectral Matches (#)			
				TP	6 kDa	7 kDa	14 kDa
Dbfp1-f1	WNDKYPGDGKDKYPGDGDDK**YLGGGNDKYLGGVFDK**YFGGGN **(42)**	4.6 kDa pI 4.4	75.07	3	1	1	1
Dbfp1-f2	KYPGDGNPKYPGGGNPKYPGGGNPKYPGGGNPKYPGGGNAK**YLGGGNDKY**P (**51)**	5.1 kDa pI 10	27.12	1	0	0	0
Dbfp2	MLSSVTLLFVACCGMAL**GQGNSWDSYRPYPVYTPKPSYPDYPEKPYPPKQTYPTYPEKKYPTYPEKK**YPTYPEKKYPTYPEKK**YPTYPEKKYPTYPEKK**YPTYPEP**TYPTYTEKKYPAYTPK**TYPTYTEKK**YPDYPEKK**YPDYPEKKYPDYPEK**KYPSYPEKKYPAYPPKNSYPGRYPW**RR **(164)**	20.2 kDa pI 9.7	90.13	16	4	6	4
Dbfp4	MFGLVAVSVFLFCHSSA**FSNTWQNR**IK**QRPTPVVPF**K**LEWYLGKWFTQSRQEPCSWKGSADFENMELNFVLDPKKNILYDHSIWKK**NNR**CVFVTFDIIPSPK**TPGTFLIQDPLGDIQSGEYVILAIDPCK**FVVEWGCTKPSPIGQRCDDPWVSVHTRERPSPKVLAEVDLALMR**TVGVRLAELPR**LSHANTPCCLGEGKLIQHDFL (190)**	21.8 kDa pI 8.1	149.48	20	0	0	0
Dbfb5	MFSAVTLVLLVSCCGTALSQRNSYGNYRPVKPPGQPINQYNQYSNPYRPQYNQNWNPYRPEQAPR**YPQQSYPAYPPK**QPYPAYPTKQPYPTDPPKQPYPANPSKPSYPANPPYDPCDEVYCRPIYCPNGQYKPTGECCPQCQPGTYLPKPWSWRGQGNVVGEQEK**FVGEGNVVGDQTYDVGGQGNVVGGQR**NVVDGKGNVVGEQRNNVGG **(190)**	21.3 kDa pI 8.7	85.71	3	1	0	0
	Dbfp5β variant MW:15.6 kDa, pI: 5.7						
Dbfp6	MFSAASFLLLVMFCGTVTS**QFYWGYLPQRLYPRDPCDDVDCRTPHCPNGGYIPIGQCCPK**CKPAASWALEVTLHVFSGRPDPEYVIPR**DTSAYDAILKAIGDTSTPLGER**LGYNGFTVIQTHGDSEVSHWTVGWCTRPKVELR**LLAAVSAMTPIGDQHPLQKEVIDTVKQSIMLCKV (154)**	17.6 kDa pI 6.6	110.57	9	1	1	1
Dbfb7α	MFFAVTLVLLVSCSGTPLGK**WDPYGSSYGNSYGRPYGKAFNPYNQYGNSYPQNNQKWNSYWPNYKQPWNSYGPQQYPSYPQ**SGSYYPGSWGWPGNNVGSQGN**AVDGLWNVVGWQGNDVDGLGNNVGKQWNDVDGVGNYVGKQWNNVD (132)**	13.2 kDa pI 4.7	147.16	16	10	10	9
	Dbfb7α–Dbfb7ζ variants MW/pI: 14.8/6.6, 14.2/4.3, 13.2/4.7, 12.3/4.7, 10.3/8.7, 9.7/9.1						
Dbfp8β	MKLALLAVIAFVAPSCYEATYPVPNQGRCLKDGQYFASGH**FVDPTNR**CTSCECFPGGNYQCRR**DACPALSCPVNQRFYPHDACCQR**CHGVIHSPGSASSVSSSDHDTSGTSRHTSKSSKSSRGTSKNSKSSKKSSKSKSSRKGSKGSRKGSRKGSRKGYKKYGKKGSGSSS **(152)**	16.3 kDa pI 10.6	68.3	3	0	0	0
	Dbfp8α variant MW,pI: 16.7 kDa, pI 10.7						
Dbfp9β	MNTKQLMCLLVAAALLLASAPAANA**RFVYGDYDDDYGYGGKYG**YPGNYGYPGNSGYPGNYGGYGNYGDNDY*GGWLGGLLGGGGRGNKWGGNYGNYGYGK **(75)**	7.9 kDa pI 4.6	106.28	9	9	7	1
	Dbfb9α variant MW,pI: 9.0 kDa, pI 4.3						
Dbfp10α	MQSAVTLLLLVSCCGMALGQWDDY**DDWDWPTGYPSYPPKQSYPPYPPYDPCKNVNCIQVVCPYGEYTPPGK**CCPVCIDWGWPWGPYGSSGSDDYDDDDDYWPYNWGK **(88)**	10.3 kDa pI 3.5	101.96	4	0	0	0
	Dbfb10β and Dbfp10γ variants MW/pI (kDa/pH): 7.9/4.7, 6.6/8.6						
Dbfp11γ	MCSATPFLLLVTFCGAVSS**LWYPDRPCYGKVCPAIYCLYGQVTPPGK**CCPQCKPDPGSNVHVPCKK**DKDCAYVVCENPGEKVECHDAPSSYPPRR**ECHCHIPEECEKDFDCVDECGPGATCDDGACHGNDCDHT (**115)**	12.7 kDa pI 4.4	137.49	0	0	0	0
	Dbfb11α–Dbfp11ε variants MW/pI (kDa/pH): 13.1/5.5, 12.7/4.4, 12.5/4.9, 12.4/4.9						
Dbfp12δ	MALSTWSLFLIVAATMYTGSCQECPVGSILKGCEFIQGHEGPWCPVGYYCRDLMMNNLGICCR**NVCWDGPPITDNYGR**AIDCSRGR**TGLCPGATECVR**YGRYGAR**SFCCNIR**VTIG **(96)**	10.5 kDa pI 7.8	119	3	0	0	3
	Dbfp12α–Dbfp12δ variants MW/pI (kDa/pH): 10.9/8.3, 10.7/8.1, 10.7/8.1						
Dbfp13α	MKGVFLLLAIVCIMVEAGGRRNQRPMYRRRLPPPTTKKPPPRPTPAPTQAPR**GGPYEHEQLTQNIEK**QLKEMNTTLDAIYTLTNEMFTIRNKCLDAYRPSG **(82)**	9.6 kDa pI 10.8	52.4	2	0	0	0
	Dbfp13α–Dbfp13ε variants MW/pI (kDa/pI): 9.6/10.8, 9.2/10.6, 8.3/10.2, 7.1/9.94, 7.1/10.0						
Dbfp14γ	MGPNKLFVTVLILCLMGAM**GGSDDPPAAPGR**CYCR**RVCRPNECYVGR**CPRRR**SWSWCCHR**SHPDCEGK **(49)**	5.6 kDa pI 8.6	89.84	6	6	14	7
	Dbfp14α-Dbfp14δ variants MW/pI (kDa/pH): 6.6/8.2, 6.5/8.6, 5.6/8.6, 5.4/8.2						
Dbfp15α	MCLAAAVLLAIAPIANAKYGSSSDSSDSDGYNGKRGGYGR**RGGLPWPR**YGRGK**GYGGWGDNYGAVPT**YGK **(52)**	5.5 kDa pI 9.9	69.24	2	0	2	1
	Dbfp15β variant MW/pI (kDa/pH): 5.0/9.0						
Dbfp16	MFSAVVTVLIICLMGVMGGGDREPAGPDWWCDWRWQCSR**NECVVFEDFGGKFCCLWTSPLCWSKD**R **(44)**	5.3 kDa pI 4.4	68.66	3	1	1	0
Dbfp17	MTSVRILVVLMVVCILAGSVVQQAEAQCHMTLK**GCANNECFTGTVGK**RKKCCPK**KNNACPQGLPSV (44)**	4.3 kDa pI 9.2	47.92	2	0	0	0

Novel proteins have been named Dbfp4 – Dbfp17. Signal peptides are underlined and matching peptide spectra are indicated in bold. For proteins with isoforms, the transcript with highest number of observed spectra or the largest molecular weight is shown as a representative sequence. Proteins variants are listed beneath the representative sequence.

^a^Sequence properties were calculated after removing the predicted signal peptide sequence.

^b^N and Q signify asparagine deamidation, and Y signifies tyrosine hydroxylation, respectively.

^c^PEAKS scoring method: a −10LogP score cut-off of 20 is equivalent to P-value of 0.01.

We report here novel sequence data for fourteen new proteins, Dbfp4 to Dbfp17, and two previously known quagga mussel byssal proteins^13^, Dbfp1 and Dbfp2 (Table 2). Quagga mussel proteins that displayed homology to zebra mussel proteins were designated with the same foot protein number to keep the *Dreissena* species foot protein nomenclature consistent. Novel quagga mussel proteins without zebra mussel homologs were named sequentially in decreasing order of molecular weight. Many of the proteins exhibit polymorphism. The protein variants all have similar primary structure but different molecular weights and isoelectric points. Most of the protein variants were clustered by Mind Trinity in the same library component. Variants are labeled with Greek letters α, β, γ, etc. by decreasing molecular weight. Including variants, 48 protein sequences are reported (Supplemental Table S3). Either the largest transcript or the transcript with the highest observed number of spectra, is listed as a representative sequence to represent the protein family in Table 2. Start codons, signal peptides, and stop codons were observed for nearly all proteins. Some transcripts contained incomplete signal peptides; variants from the same protein component were used to complete signal peptides where possible (Supplemental Table S4). Proteins ranged in size from 4.3–21.8 kDa, consistent with our previous MALDI-TOF study on secreted byssal proteins^15^.

Collectively, the novel byssal proteins were abundant in proline (P), glycine (G), tyrosine (Y), and have significant amounts of cysteine (C), lysine (K), asparagine/aspartic acid (N/D), and arginine (R) (Table 3). Glutamine (Q) and tryptophan (W) were also observed in high relative abundance in select proteins. Byssal proteins were categorized according to their amino acid content as ‘Glycine and Tyrosine rich’ (G, Y rich), ‘Proline rich’ (P rich), and ‘Cysteine rich’ (C rich). One protein did not fall into the above categories and is described separately. A range of glycine and tyrosine rich proteins are also found in the marine mussel, *Mytilus californianus*, foot transcriptome^28^, many of which are associated with the byssal-substrate interface. Like quagga mussels, the *M. californianus* byssus also contains cysteine rich proteins; in the latter these are associated with the cuticle of the byssal thread. The quagga mussel transcriptome has more proline rich proteins and a lack of histidine and lysine rich proteins in comparison to the *Mytilus californianus* transcriptome. Spatial characterization of the quagga mussel foot proteome is needed to confirm whether amino acid composition relates to byssal localization in these mussels.

**Table 3:** Prominent amino acids in quagga mussel byssal proteins and their respective protein category.

Protein	Prominent amino acids (mol%)				Protein category
	1^st^	2^nd^	3^rd^	Notable	
Dbfp1^a^	G (26.6)	Y (14.7)	P (12.2)	K (12.3)	G, Y rich
Dbfp7α	G (18.6)	N (13.6)	Y (10.2)	Q/P (9.2)	G, Y rich
Dbfp9β	G (37.8)	Y (21.6)	N (10.8)		G, Y rich
Dbfp15	G (30.8)	Y (13.5)	S (11.5)		G, Y rich
Dbfp2	P (25)	Y (23.2)	K (17.7)	T (10.4)	P rich
Dbfp4	P (8.9)	L (8.9)	V (7.4)		P rich
Dbfp5	P (18.4)	Y (11.1)	G (11.1)	Q (10.5)	P rich
Dbfp6	P (9.5)	G (7.6)	L (7.6)	V (7.6)	P rich
Dbfp10α	P (18.2)	D (15.9)	Y (13.6)	C (6.8)	P rich
Dbfp13α	P (15.9)	R (12.2)	T (12.2)	Q (6.1)	P rich
Dbfp11γ	C (15.7)	P (13.0)	D (10.3)	Y (5.2)	C rich
Dbfp12δ	C (12.5)	G (11.5)	R (10.4)	P (8.3)	C rich
Dbfp14γ	R (18.4)	C (16.3)	P (12)	G (10.2)	C rich
Dbfp16	C (13.6)	W (13.6)	P (6.8)		C rich
Dbfp17	C (15.0)	K (15.0)	G (10.0)		C rich
Dbfp8	S (22.7)	L (12.0)	G (11.3)		—

^a^Rzepecki and Waite 1993b^12^.

RSEM analysis software was used to estimate gene and transcript abundance from the RNA-sequencing data. To provide a condensed overview of expression of quagga mussel byssal proteins, the expected expression for each protein gene is listed, not each individual transcript (Table 4). Expression rank was determined by sorting by expected count.

**Table 4:** Expression analysis of quagga mussel byssal proteins.

Protein	Transcriptome component	Expected count	Transcripts per million (TPM)	Fragments per million (FPM)	Rank
Dbfp9	comp42199_c1	31302209	130366	252497	2
Dbfp14	comp35860_c0	18547819	51358	98942	4
Dbfp9-start	comp31072_c0	14439051	71373	137830	5
Dbfp7	comp52765_c1	10955530	12930	25112	7
Dbfp9-alt-start	comp31079_c0	9617925	68934	133137	9
Dbfp2 middle	comp12693_c0	9558067	48823	94269	10
Dbfp11	comp49590_c4	9360859	13623	26269	12
Dbfp1-fragment	comp39272_c0	9347159	83646	162151	13
Dbfp2-end	comp31091_c1	5283169	21456	41387	16
Dbfp2-start	comp9618_c0	5014310	27942	53928	18
Dbfp10α/β	comp35857_c0	4541260	3914	7545	22
Dbfp1-start	comp34314_c0	4163681	30984	60032	25
Dbfp1-middle	comp9617_c0	2604072	23918	46162	30
Dbfp15	comp37514_c0	2561050	7518	14572	31
Dbfp4	comp37554_c0	1999121	1957	3745	35
Dbfp10c	comp40957_c0	1487483	2738	5323	44
Dbfp9η/θ	comp35873_c0	1348059	2258	4381	51
Dbfp1-end	comp30958_c0	1103356	4990	9635	60
Dbfp9α	comp58518_c0	769975	3094	6074	76
Dbfp13α-δ	comp49016_c0	640379	656	1270	82
Dbfp17	comp12176_c0	814345	1572	3022	103
Dbfp16	comp13545_c0	436715	2001	3876	120
Dbfp6	comp31337_c0	143463	192	368	141
Dbfp5	comp47359_c0	309503	46	90	172
Dbfp13ε	comp57838_c0	199337	32	62	259
Dbfp12	comp45612_c1	62698	81	152	675

### Glycine- & tyrosine- rich proteins

#### Dbfp1 – Improved cuticle protein primary sequence information

Dbfp1 has been previously identified and partially sequenced; isolated Dbfp1 has a mass of a 68 kDa peak (by MALDI-TOF MS) and contains DOPA (typically 0.5 mol% up to 2.8 mol%)^13^. Two components were identified using LC-MS/MS that resemble Dbfp1 fragments, named Dbfp1-f1 and Dbfp1-f2 (fragment 1 and 2). Dbfp1-f1 contains the previously observed consensus sequence DKYFGGGN observed by Anderson and Waite^21^. The second identified component Dbfb1-f2 contains an octapeptide sequence PKYPGGGN repeated 4 times consecutively, similar to the consensus sequence DKYPGGGN. Interestingly, none of the observed spectra contained tyrosine hydroxylation modifications. It is possible that DOPA-containing peptides cross-linked in the whole TP extract, and therefore could not be denatured or accessed by trypsin. Using known Dbfp1 sequences identified by Rzepecki and Waite we were able to mine additional Dbfp1 sequence data from the library, including the Dbfp1 start codon, signal peptide, and end codon (Supplement Table S4). Although the entire Dbfp1 sequence could not be fully assembled, the essence of the protein has been revealed: the N-terminus, the central repeated domains, and the C-terminus. The role of Dpfp1 in the zebra mussel byssus is thought to be either as a cuticle varnish, or a structural cohesive component^53^. Dbfp1 is believed to be the quagga mussel homolog of Dpfp1 and by extension may serve a similar function in the byssus^13,21^.

#### Dbfp7 – Polymorphic byssal protein containing DOPA

**Figure 1:**
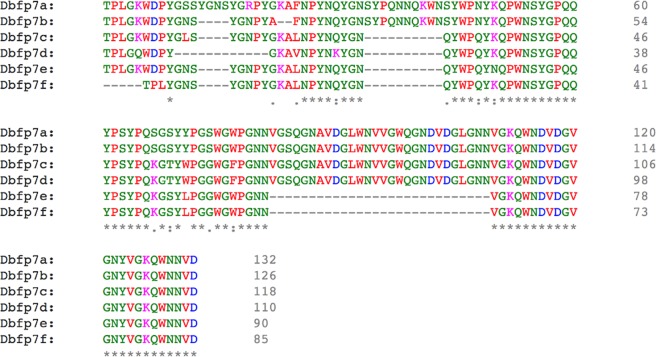
Dbfp7 variants with signal peptide removed, aligned using Clustal to illustrate the effects of alternative splicing. Greek letters replaced by English letters. Stars beneath sequences indicate exact match, a colon dot indicates substitution with strongly similar properties, and a single dot indicates a substitution with weakly similar properties.

Six variants of Dbfp7 were identified in all samples with theoretical masses ranging from 9.4–14.8 kDa, shown in Fig. 1. Of the newly identified byssal proteins, Dbfp7 had the highest spectral count in whole TP extract and in the 6–14 kDa bands. Unique spectra were not observed for each variant, likely due to their similarity in their primary sequence. Thus, we cannot confirm each transcript exists at the protein level. However, RSEM analysis suggests that the reported variants are all expressed, and thus all variants observed by LC-MS/MS were listed (Supplemental Table S3). RSEM analysis (Table 4) predicted Dbfp7 as the 7th-most abundantly expressed gene in the quagga mussel foot transcriptome, and the 3rd most abundantly expressed byssal protein gene.

Dbfp7 displays significant homology only to the zebra mussel Dpfp7, with expect values for the three zebra mussel proteins Dpfp7 variants of 7e^−39^, 4e^−34^, and 2e^−27^, respectively. Homology between species of the same family has also been observed between members of *Mytilus* marine mussel family^9^. Dbfp7 is rich in glycine (18.6%) and tyrosine (13.6%), which are also abundant in both marine mussel and zebra mussel byssal proteins^54,55^. In the supporting spectra from the whole TP extract in LC-MS/MS, a single tyrosine hydroxylation in Dbfp7γ, Dbfp7δ, and Dbfp7ζ was observed in the same position. Previously, Rzepecki and Waite identified a faint series of bands appearing at approximately 12–13 kDa using a DOPA-specific stain; these bands were labeled as the quagga mussel homologue of Dpfp3^13^. The Dbfp7 family includes variants within 12–13 kDa, and Dbfp7 appears to include at least a single DOPA molecule; it is likely that Dbfp7 is actually the quagga mussel homologue of Dpfp3 previously identified. Due to the significant homology between Dbfp7 and Dpfp7^14^, the new quagga mussel proteins have been named as such to keep the protein naming convention consistent within the *Dreisennid* species. The most intense staining observed from induced byssal proteins separated by SDS PAGE was the 6/7 kDa doublet: Dbfp7 appears to be the most abundant protein in these gel bands, and by extension must represent a significant byssal protein. Although Dbfp7 has not been specifically localized within the byssus, the results suggest the protein is an abundant byssal component, and thereby could be a structural cohesive component or potentially have surface adhesion role.

#### Dbfp9 – Polymorphic byssal protein

Two Dbfp9 variants were observed with assembled theoretical masses of 7.9 and 9 kDa. Dbfp9 was separated over multiple components. One transcript contained the complete signal peptide and N-terminus, which was the fifth most abundantly expressed transcript according to RSEM analysis. A separate component contains multiple variants of the C-terminus of Dbfp9; this was the second-most expressed component in the library according to RSEM (Table 4). Dbfp9 was manually assembled using additional *de novo* assembled spectra (Supplement Table S5). Mining the library using the component numbers fingerprinted by LC-MS/MS, additional C-terminus transcripts variants were found. These protein variants were identified by LC-MS/MS however their transcripts were shorter and did not overlap with the N-terminus of the protein, and therefore could not be accurately assembled. However, aligning these additional transcripts to the longest Dbfp9 C-terminus transcripts suggests that the Dbfp9 variants could range in mass from 6.6–9.0 kDa (Supplemental Fig. S3). Dbfp9 had the second highest number of spectra in the 6 kDa band, which roughly matches its assembled mass. BLAST analysis against the zebra mussel foot library revealed homology to the zebra mussel Dpfp9 variants^43^.

Dbfp9 has the highest abundance of glycine (38%) and tyrosine (22%) observed in the reported quagga mussel proteins, and asparagine (10%) is also abundant. This is a characteristic that has been observed in the marine mussel adhesive protein Mfp-3^54^. An interesting tandem repeat (Y/S)GYPGN was observed three times in Dbfp9β, with tyrosine being highly conserved. Other interesting motifs include the sequence GNYG, observed five times in Dbfp9β. Dbfp9α contains a triple-tandem repeat of GGNY at the N-terminus. One variant observed with one spectra in the 7 kDa band contained the sequence NYGYPGYGG repeated five times, similar to the consensus sequence found in a marine mussel thread matrix protein, TMP-1: GYGYGNYGYGY^56^. GYG is also a defining feature of keratin-associated proteins, pearl oyster shell matrix, and shematrins^36^. However, the transcript was incomplete (no start or end codon) and was not included in the variant list. The C-terminus of Dbfp9 contains the sequence Y*GGWLG(G/Q)LLGGGG(R/K)G, where the first tyrosine may be hydroxylated to DOPA; only one supporting spectrum was observed from all samples that contained a tyrosine hydroxylation at this position. The C-terminus of the zebra mussel homolog Dpfp9 has a similar motif with four glycine residues in succession. Calculating the isoelectric point using the conserved C-terminus sequence continued to the stop codon results in a basic pI, also observed in zebra mussels. The N-terminus of Dbfp9, RFVYGDYDDDYGYGG, has a distinctly acidic isoelectric point of 3.4, also observed in the zebra mussel homologs. This block structure of N- and C-termini with opposing pI is present in several byssal proteins.

#### Dbfp15 – Small, highly charged byssal protein with high serine content

Dbfp15α was identified in the whole extract, 6 kDa band, and 14 kDa band by the same spectra match, and was identified by two spectra matches in the 7 kDa band. Only one spectrum was identified for Dbfp15β, however it was included due to being part of the same component as Dbfb15α. The masses of Dbfp15α and Dbfp15β are 5.5 and 5.0 kDa, respectively. Dbfp15α and Dbfp15β both contained part of the signal and were predicted for excretion, however they lacked start codons. A third variant in the library Dbfp15γ with mass of 5.8 kDa was not observed in the LC-MS/MS data, however, Dbfp15γ contained an intact signal peptide with a start codon. The signal peptide overlapped exactly with partial signal peptides from Dbfp15α and Dbfp15β, and thus was used to complete their respective N-termini.

Dbfp15 has the second highest relative abundance of both glycine (31%) and tyrosine (14%) of the reported proteins, similar to Dbfp9. Dbfp15β has two instances of tyrosine adjacent to multiple glycine residues, in the form of GGYGG and GYGG, similar to Dbfp9. Dbfp15α and β have isoelectric points of 9.9 and 9.8 respectively, resulting in a net +4 charge at neutral pH. Both variants at the N-terminus contain the sequence YGSSSDSSDSDGY, notable due to its abundance of serine clusters, aspartic acid and tyrosine. In sandcastle worms, the adhesive mechanism involves highly phosphorylated serine residues^57,58^. However, no direct spectral coverage of phosphorylated serine residues in Dbfp15 were observed. The highly conserved N-terminus is acidic with a pI of 3.3.

### Proline-rich proteins

#### Dbfp2 – Sequence expansion of the DOPA-containing block-structure protein

The Dbfp2 sequence was assembled from 4 different components observed by LC-MS/MS from the whole TP extract, named Dbfp2-f1 through Dbfp2-f4, with an assembled mass of 20.3 kDa (Supplement Table S6). Dbfp2 was first identified by Rzepecki and Waite using gel electrophoresis with an estimated mass of ~22 kDa, similar to the theoretical assembled mass^13^. BLAST results of the assembled Dbfp2 protein against the zebra mussel Dpfp2 produced an expect value of 9e^−17^, suggesting that Dbfp2 is likely a homolog of Dpfp2^13^. The component that contains the central repeated domains of Dbfp2 was predicted to be highly expressed, the 10th most abundant gene in the transcriptome (Table 4). Previous amino acid analysis of Dbfp2 revealed a high abundance of proline (23%), tyrosine (21%), lysine (15%), and threonine (9%), and small amount of DOPA (2.2%)^13^. Our assembled Dbfp2 theoretical amino acid content is similar: proline (25%), tyrosine (23%), lysine (18%), and threonine (10%). No tyrosine hydroxylations were observed by LC-MS/MS; this could be a result of less DOPA in induced proteins compared to naturally secreted proteins. Alternatively, the DOPA-containing portions of the protein may be cross-linked, preventing trypsin digestion prior to LC-MS/MS^59^.

**Figure 2:**
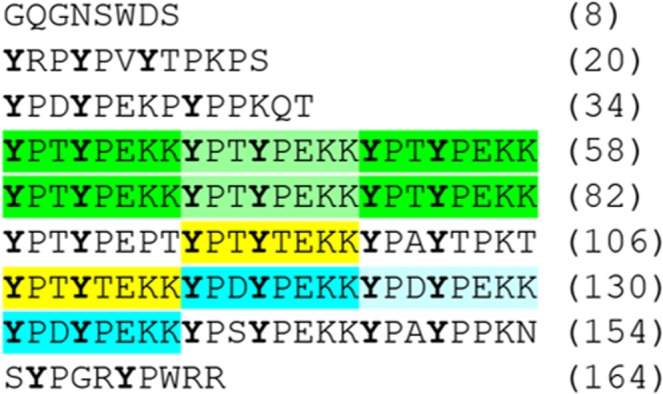
Dbfp2 assembly, with tandem repeats highlighted in green, yellow, and blue.

Dbfp2 has a highly repetitive block structure. The Dbfp2-f2 transcript contains an octapeptide sequence YPTYPEKK consecutively repeated five times (Supplement Table S6). *De novo* assembly during the transcriptome construction likely could not fully assemble the protein because the highly repetitive YPTYPEKK domain at the DNA level is beyond the resolution of the 150 bp paired-end Illumina sequencing. Dbfp2-f1 contains the signal peptide and start codon and ends with a single YPTYPEKK sequence. Dbfp2-f3 has the same motif of YPTYPEKK observed four times, highlighted in green in Fig. 2. The Dbfp2-f3 C-terminus contains two repeats of the sequence YPTYEKKY (yellow), and triple tandem repeat of the sequence YPDYPEKK (blue). A consensus sequence combining both of these motifs is YP(T/D)Y(P/T)EKK, where the tyrosine, proline, lysine, and glutamic acid residues are highly conserved. Tyrosine positioning throughout the central region Dpfb2 is highly conserved, with the pattern YxxYxxxxY, where x represents any amino acid.

#### Dbfp4 – Largest novel byssal protein with heterogeneous amino acid composition

**Figure 3:**
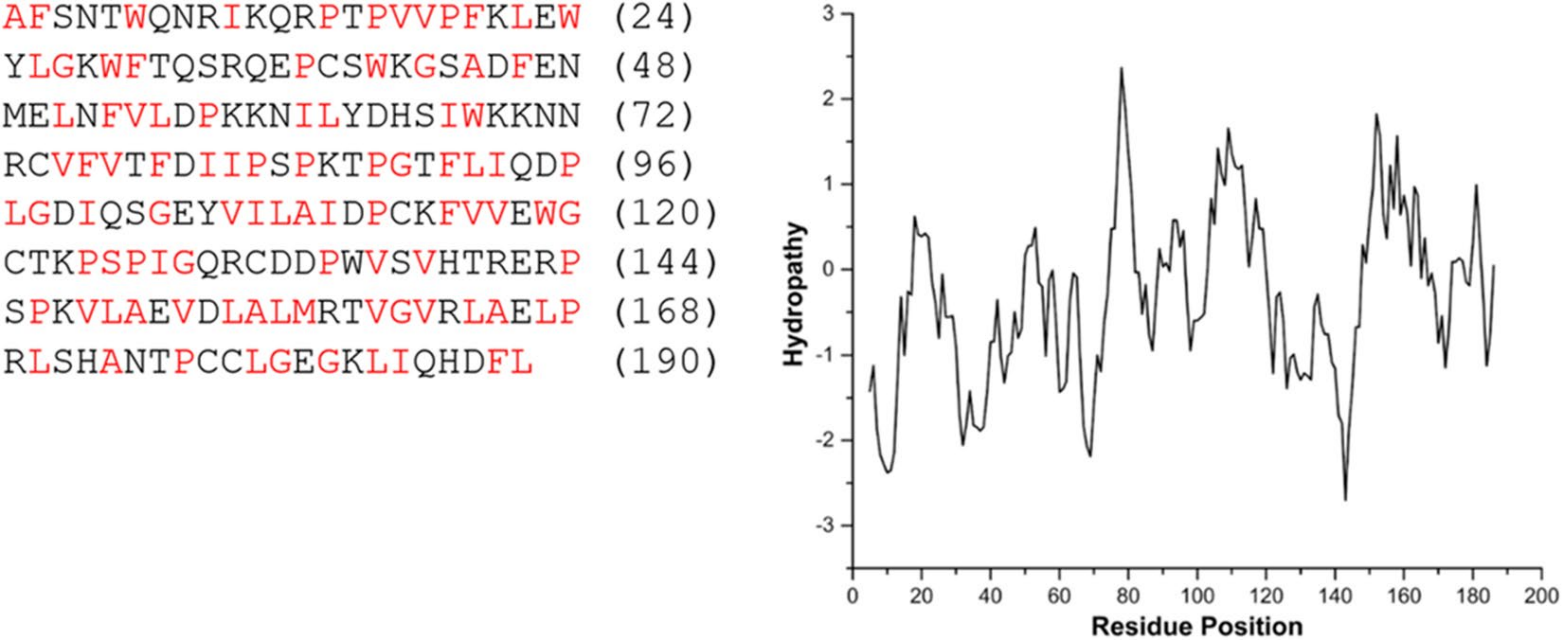
Kyte-Doolittle hydropathy plot (right) for Dbfp4 indicating alternating domains; higher score indicates higher hydrophobicity. Hydrophobic residues are highlighted in red (left).

Dbfp4 was identified with 76% coverage by 20 spectra in the whole TP extract and has an estimated mass of 21.8 kDa and an isoelectric point of 8.7. Dbfp4 has very heterogeneous amino acid composition with no residue present above 10% (Table 3). While most marine and freshwater byssal proteins contain dominant amino acids (>10 mol%), some marine mussel byssal proteins putatively assigned to the bulk plaque and cuticle (Mcfp-10, -15, and -17) are also heterogeneous in amino acid composition, which suggests that Dbfp4 has similar protein localization and function^28^. BLAST results of the protein reported minor homology to titin-like isoform; it is possible Dbfp4 could provide passive elasticity to the quagga mussel byssus as titin provides elasticity to muscle tissue, although titin is considerably larger than Dbfp4^60^. Notably, Dbfp4 has alternating hydrophobic and hydrophilic domains (Fig. 3), also observed in the ECM protein elastin, which could play an important role in its structure and function^61^.

#### Dbfp5 – Byssal protein with significant homology to zebra mussel byssal protein Dpfp5

Dbfp5 has a molecular mass of 21.3 kDa and is a basic protein with an isoelectric point of 8.7 and a net charge of +4.2 at pH 7. Dbfp5 was identified by three spectra in the whole TP extract, and one spectrum in the 6 and 7 kDa bands. Dbfp5 is clearly a homolog of the zebra mussel Dpfp5, with a BLAST expect value of 2e^−66^. Dbfp5 is rich in proline (18.4%), tyrosine and glycine (11.1% each), and there also are significant amounts of glutamate (10.5%) and valine (7.9%).

**Figure 4:**
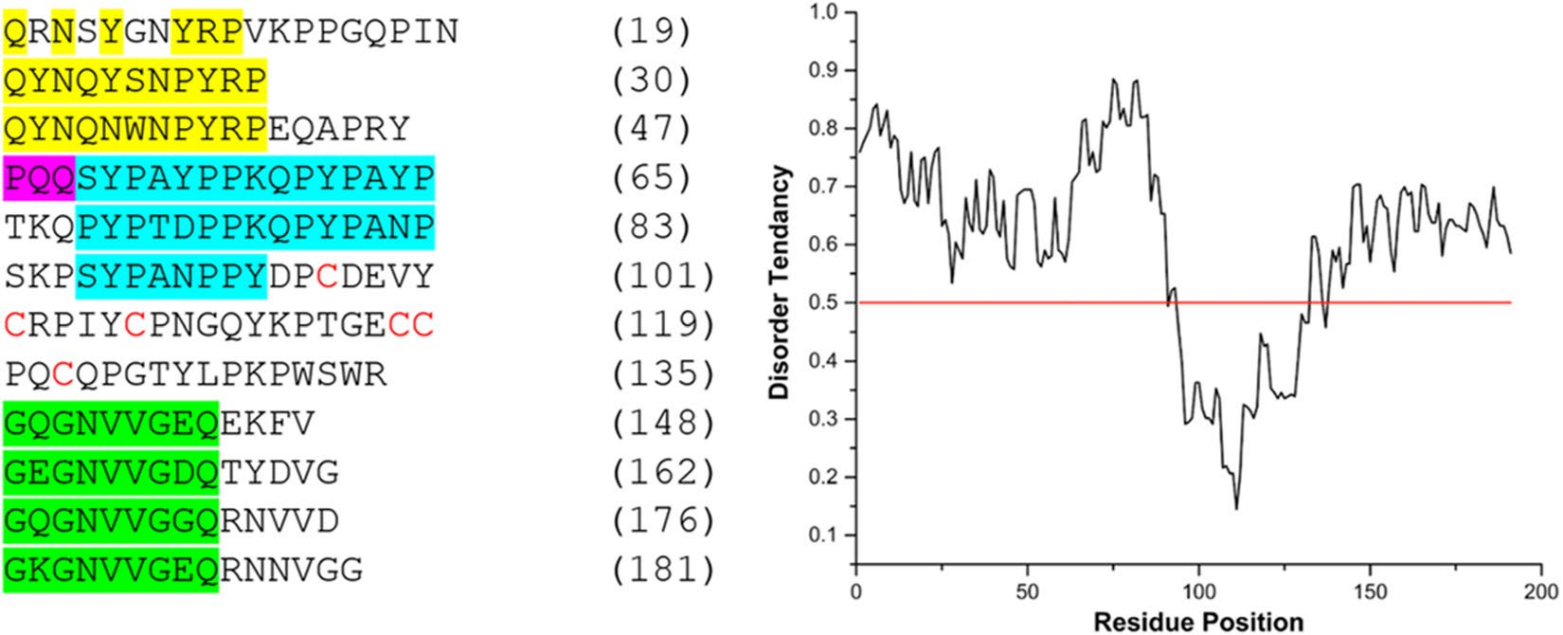
Dbfp5 protein with interesting repeats highlighted (left), and IUPred disorder prediction chart for Dbfp5, where values above 0.5 indicate a tendency to be disordered (right). Note how the cysteine-containing region has low disorder.

At the N-terminus of Dbfp5 there is a tandem repeat of QYNQ(Y/N)(S/W)NPYRP, highlighted in yellow in Fig. 4. There is only one instance of a PQQ repeat, unlike Dpfp5 which contains multiple PQQ and PKQ repeats^43^. The central region of the protein contains two instances of the sequence (S/P)YP(A/T)(Y/D)PPKQPYPA(Y/N)P; this sequence is rich in aromatic resides, and is similar to the SYP(A/T)YP repeats in Dpfp5. Using IUPred Dbfp5 is predicted to be disordered in the repeated N-terminus region (Fig. 4). The central region of Dbfp5 contains six cysteine residues (highlighted in red), which could confer interior protein stability through disulfide bonding. This notion is supported through disorder prediction, which suggests the cysteine-containing region of the protein has low disorder tendency.

The C-terminus of Dbfp5 contains four repeats of G(Q/X)GNVVG(E/Z)Q, where X can be lysine or glutamic acid, and Z can be glycine or aspartic acid. Notably, Dbfp5 contains VGG and VGD motifs also found in Dpfp5; motif conservation within the species may suggest it is integral for protein function. Interestingly, VGG repeats are also observed in the sandcastle worm glue protein, Pc-1^36^. The repetitive C-terminus of Dbfp5 is also predicted to be disordered and is acidic with a pI of 4.2.

#### Dbfp6 – Byssal protein with heterogeneous composition

Dbfp6 was identified in the whole TP extract by 3 spectra, with a theoretical mass of 17.6 kDa and an isoelectric point of 6.6. BLAST analysis of Dbfp6 produced no significant match to the zebra mussel foot library. Dbfp6 has very heterogeneous amino acid composition, similar to Dbfp4 with no residue present above 10%. Significant amino acids include proline (9.5%), with equal parts of glycine, leucine, and valine (7.6%). Dbfp6 also displayed alternating hydrophobic and hydrophilic domains similar to Dbfp4, which could play an important function to protein function and folding. Dbfp6 contains two cysteine doublets located at the start and end of the sequence, and the remaining cysteine residues are evenly spaced throughout the sequence. The N-terminus of the Dbfp6, including the first cysteine doublet, produced weak BLAST hits to collagen and ECM kielin/chordin-like proteins found at the first cysteine doublet, suggesting Dbfp6 may have a structural role or provide elasticity to the byssus.

#### Dbfp10 – byssal protein with variable proline and tyrosine content

Three variants of Dbfp10 were observed, with masses of 6.6, 7.9, and 10.3 kDa respectively. Dbfp10α was observed only in whole TP extract, whereas Dbfp10β and Dbfp10γ were observed in the gel bands. Dbfp10α is rich in proline (18.2%), asparagine (15.9%), tyrosine (13.6%), and notably high amounts of tryptophan (9.1%) and cysteine (6.8%). BLAST produced weak hits to kielin/chordin-like extracellular matrix (ECM) proteins in a variety of species with the proteins aligning to regions containing cysteine residues, such as YTPGKCCPVC, which suggests Dbfp10 may have an ECM binding role or structural role in the byssus. Dbfp10γ displays significant homology with Dpfp10 in zebra mussels, with a BLAST expect value 5e^−28^. The C-terminus of Dbfp10α and Dbfp10δ each have a cluster of negatively charged residues and aromatic residues: DDYDDDDDYWPYNW, which is a characteristic of non-collagenous proteins involved in metal binding and mineralization^62^.

#### Dbfp13 –intrinsically disordered byssal protein

Eight variants of Dbfp13 were identified, with masses ranging from 7.1–9.6 kDa, and isoelectric points ranging from 9.9–10.8. Dbfp13α and Dbfp13β have the highest isoelectric point observed of the reported proteins at a pH 10.8. Dbfp13α to Dbfp13δ were identified by 2 spectra and are located within the same component. Dbfp13ε was identified by three unique spectra and is from a separate library component. Many of the Dbfp13 variants share the same identifying peptide spectra, thus we cannot confirm their protein-level presence, however gene expression analysis suggests all of the variants were significantly expressed.

**Figure 5:**
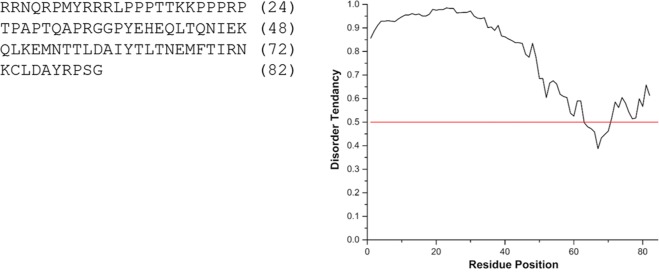
IUPred Disorder prediction chart for Dbfp13α, where values above 0.5 indicate a tendency to be disordered.

The N-terminus of Dbfp13 is abundant in proline and arginine, with Dbfp13ε containing a triplet of arginine, two triplets of proline, and a doublet of lysine: RRRLPPPTTKKPPPRPTP. Similarly, Dpfp13α contains a doublet and triplet of arginine, followed by two proline triplets and a lysine doublet: RRNQRPMYRRRLPPPTTKKPPPRP. The local abundance of lysine and arginine results in a very basic N-terminus with an isoelectric point of 13.0. Disorder prediction of Dbfp13α suggests the N-terminus is intrinsically disordered; Dbfp13 had the highest disorder tendency of the new QM proteins (Fig. 5). Protein disorder could be important to protein function, as it could enable the protein to form many different physical conformations to interact with a variety of surfaces. The high isoelectric point and disorder tendency suggest that the N-terminus of Dbfp13 could have an active intermolecular role.

### Cysteine-rich proteins

#### Dbfp11 and Dbfp12 - byssal proteins with regularly spaced cysteine and lysine residues

Dbfp11 is acidic with isoelectric points varying from 4.4–5.5, whereas Dbfp12 isoelectric points are basic, varying from 7.8–8.3. Dbfp11 contains the most cysteine (15.7%) of the novel quagga mussel proteins along with significant amounts of proline (13.0%), aspartic acid (10.3%), and glycine (9.1%). Dbfp12 also is rich in cysteine (12.5%), glycine (11.5%), arginine (12.2%), and proline (8.3%). Both Dbfp11/12 have proline and cysteine distributed evenly throughout the protein sequence, with proline and cysteine doublets both observed. In marine mussels, Mefp2 contains a significant amount of cysteine and is thought to have a structural role in the plaque foam^62^. Dbfp11/12 did not produce BLAST results to known zebra mussel proteins.

#### Dbfp14

Four variants of Dbfp14 were observed in the whole TP extract, with theoretical weights ranging from 5.4–6.6 kDa, and isoelectric points ranging from pH 8.2–8.6. Dbfp14 was the fourth-most expressed gene in the foot transcriptome library and was the second most abundant protein in the 7 and 14 kDa gel band LC-MS/MS data. Dbfp14 is rich in arginine, however variants such as Dbfp14α have a notably high tryptophan content (13.0%). BLAST results of Dbfp14 produced no hits to known zebra mussel proteins, and no significant hits to other known proteins. Dbfp14δ contains two cysteine doublets, and cysteine spacing and position is generally conserved between variants.

#### Dbfp16

Dbfp16 was observed in the whole TP extract and in the 6 kDa and 7 kDa bands, which corresponds to its theoretical molecular weight of 5.3 kDa. Dbfp16 is one of few byssal proteins with an acidic isoelectric point of 4.4. BLAST produced no homology to zebra mussel proteins, and weak hits to tenascin isoform X3 and C (ECM proteins) in zebrafish. Dbfp16 is richest in cysteine (13.6%) and tryptophan (13.6%). Dbfp16 contains no notable repeats or motifs, however the cysteine resides are distributed evenly throughout the sequence with one cysteine doublet observed.

#### Dbfp17 – Small byssal protein with high lysine & cysteine content

Dbfp17 was observed only in the whole TP extract by two spectra and has a theoretical mass and isoelectric point of 4.3 kDa and pH 9.2, respectively. The identification score for Dbfp17 was below the criteria of acceptance with a −10LogP of 48, however it was included due to the theoretical weight of Dbfp17 aligned with a MALDI-TOF peak observed with notably higher intensity in the plaque spectra^15^. BLAST did not produce any significant hits to known proteins in the zebra mussel and other species. Dbfp17 is rich in both cysteine and lysine, (15%), and has a notable relative amount of glycine (10%), proline (7.5%) and threonine (7.5%). There is one notably hydrophilic region in the center of the protein sequence that contains multiple lysine residues and a pair of cysteine residues: KRKKCCPKK. This region may be exposed on the outside of the protein enabling the cysteine residues to form disulphide bonds and the proline residue to interact with aromatic amino acids.

### Uncategorized protein

#### Dbfp8 – Large serine and lysine-rich protein

Dbfp8 was observed in the whole TP extract with a theoretical mass of 16.2 kDa and a basic isoelectric point of pH 10.6 similar to other byssal proteins. BLAST analysis produced no significant match to the zebra mussel foot library. Notably, Dbfp8 has the highest content of serine (22.7%) of the newly identified proteins. This raises the possibility that this protein is phosphorylated, since serine phosphorylation has been observed in adhesive proteins in sandcastle worms and marine mussels^55,58^. However, no phosphorylated serine residues were observed. Other abundant amino acids include lysine (12.0%) and glycine (11.3%), with the remaining amino acids present under 10%. Interestingly, the C-terminus of Dbfp8 contains the majority of the serine and lysine residues of the protein, including 5 serine doublets, 2 serine triplets, and 2 lysine doublets. The C-terminus is also noticeably hydrophilic and disordered compared to the N-terminus, which may be important to the protein functionality and structure.

### Evaluating the novel set of quagga mussel byssal proteins

Using SDS-PAGE to separate induced QM threads/plaques, the most stain-intense bands observed besides Dbfp1 included the ~6/7 kDa doublet and 14 kDa bands examined in this study (Fig. S2). Many of the proteins fingerprinted in this study are of a similar molecular weight to these gel-band masses. Furthermore, our group previously utilized MALDI-TOF MS to analyze the protein content of induced and isolated threads and plaques^15^. A range of proteins was observed from 4.3–18 kDa: many of the newly identified quagga mussel byssal proteins fall within this mass range observed by MALDI-TOF. The proteins we have identified and sequenced here likely represent a significant portion of the abundant and soluble proteins in the quagga mussel byssus. Many of the proteins displayed homology to zebra mussel proteins, and the foot transcriptomes are similar overall, with > 90% of zebra mussel foot transcripts from the Xu and Faisal zebra mussel foot cDNA library^42^ displaying homology to the quagga mussel foot transcripts. The byssal proteins described here are unique compared to other known proteins but do exhibit general features that are common in the proteome of other biological adhesive systems, including polymorphism, repeated sequences, disordered structure, and block structure.

#### Polymorphism

Many of the proteins display polymorphism, including Dbfp7 (10 variants), Dbfp9 (8 variants), Dbfp13 (8 variants), Dbfp11 (5 variants), and Dbfp14 (4 variants each). Polymorphism could increase the versatility of adhesion as multiple forms of an adhesive protein can interact with different surfaces^14,63,64^. Byssal protein polymorphism has been observed in both zebra mussel and marine mussel species, with proteins in a family exhibiting varied masses and isoelectric points^14,59^. The adhesive protein Mefp3 in marine mussels, for example, is estimated to have 35 variants^65^. This protein polymorphism has been hypothesized to be nature’s solution for implementing a versatile system that can interact to surfaces with different chemical properties and conformations^63^.

#### Repeated Sequences

Many proteins identified contain interesting tandem repeats or sequence patterns. A novel repeat was observed in Dbfp1, PKYPGGGN repeated four consecutive times. Dbfp2 contains a quadruple tandem repeat of YPTYPEKK within a more generally observed pattern YP(T/D)Y(P/T)EKK. The high lysine and aromatic residue content could allow for cation-π interactions with nearby tyrosine residues and other aromatic moieties^66^. Dbfp5 contains three instances of (S/Y)YP(A/T)(Y/D)PPLQPYPA(Y/N)P and four instances of G(Q/X)GNVVG(E/Z)Q, where X can be lysine or glutamic acid, and Z can be glycine or aspartic acid. The residue positioning of glycine, tyrosine, and proline are highly conserved within the consensus sequences. One variant contains the sequence NYGYPGYGG repeated five times, similar to the consensus pattern and observed in another byssal protein in marine mussels, TMP-1^56^. In general, consensus repeats are a more common feature of freshwater mussel byssal proteins than marine mussel byssal proteins. Of note, glue proteins from several other organisms also contain tandem repeats, including the terrestrial slug (*Arion subfuscus)*^26^, sandcastle worms (*Phragmatopoma californica*)^36^, and velvet worms (*Euperipatoides rowelli*)^35^.

#### Disorder

Select byssal proteins were predicted to have intrinsically disordered regions. Dbfp5 was predicted to have a disordered structure in the regions containing the previously described repeated sequences. Dbfp5 and Dbfp13 are predicted to be intrinsically disordered at the N-terminus and Dbfp8 at the C-terminus. Disordered proteins in the adhesive prey capture slime of velvet worms have been described as open, extended, with random conformations that have large surface areas and high solvent accessibility^35^. The disorder of protein structure could assist in the versatility of the proteins by increasing the chance that specific motifs would be exposed, and strong adhesive-substrate interaction would occur.

#### Block Structure

Several byssal protein sequences exhibit block-like structures. Most notably are the pI-based block structures, including Dbfp9, Dbfp15, Dbfp5, Dbfp10, Dbfp11, and Dbfp12. A distribution of charges in adhesive proteins has been described by Wang and Stewart in the adhesive system of sandcastle worms, where co-secreted adhesive components undergo electrostatic condensation in seawater to form the cured bioadhesive^67^. Another block characteristic involves regions of disorder, as in Dbfp5. As described above, disorder may assist in adhesive-substrate interactions. In the case of Dbfp5, the distinct basic N-terminus and acidic C-terminus are both predicted to be disordered and are separated by a central region of lower disorder tendency. This is an example where oppositely charged components are separated within the molecule itself rather than between proteins, a common characteristic in quagga and zebra mussel byssal proteins.

## Conclusion

Utilizing next-generation sequencing paired with LC-MS/MS, sequence information for fourteen new proteins, Dbfp4–Dbfp17, and two previously known quagga mussel byssal proteins Dbfp1 and Dbfp2 have been determined. The quagga mussel proteins display homology to zebra mussel proteins. General features of the quagga mussel byssal proteome include polymorphism, repeated sequences, disordered structure, and block structure. The reported proteins represent a significant expansion of the knowledge base of the quagga mussel byssal proteins. However, further studies are required to determine the specific roles of the different byssal proteins.

## Supplementary information

Supplemental Information

## Data Availability

Data supporting the findings of this study are available from the corresponding author on request. The transcriptome data have been deposited with links to BioProject accession number PRJDB8124 in the DDBJ BioProject database. The sequence reads are available from the DDBJ Sequence Read Archive (DRA) under the accession number DRA008242.
