# Supplementary material for: Fingerprinting of Proteins that Mediate Quagga Mussel Adhesion using a De Novo Assembled Foot Transcriptome
Source: Sci Rep. 2019 Apr 19;9:6305. doi: 10.1038/s41598-019-41976-7 (PMC6474901; doi:10.1038/s41598-019-41976-7)
Supplement: Supplementary file 1 — Supplemental Information [file 41598_2019_41976_MOESM1_ESM.pdf]

Supplementary Material

Fingerprinting of Proteins that Mediate Quagga Mussel Adhesion using a De Novo Assembled Foot Transcriptome

David J. Rees[a], Arash Hanifi[a], Angelico Obille[a], Robert Alexander[b], & Eli D. Sone[a,b,c*]

[a] Institute of Biomaterials & Biomedical Engineering; University of Toronto, Toronto, ON, Canada
[b] Department of Materials Science & Engineering; University of Toronto, Toronto, ON, Canada
[c] Faculty of Dentistry; University of Toronto, Toronto, ON, Canada

*Corresponding author. Email: eli.sone@utoronto.ca Phone: +1 416-978-7422
164 College Street, Room 407, University of Toronto, Toronto, Ontario, M5S 3G9, Canada

Contents:

1. Quagga mussel foot transcriptome library construction

- Table S1: Summary of RNA-sequencing statistics and assembly quality
- Figure S1: Distribution of transcript lengths in quagga mussel transcriptome library

2. Quagga mussel gel bands removed for LC-MS/MS

- Figure S2: Gel of QM TP Extract

3. Quagga mussel protein assembly using transcriptome library

- Table S2: Additional large and abundant proteins identified by LC-MS/MS
- Table S3: Byssal proteins identified by LC-MS/MS analysis

Dbfp1 - Mining the transcriptome library for additional fragments

- Table S4: Dbfp1 fragments and proposed overlapping sequences

Dbfp9 - Manual assembly of using de novo only spectra

- Table S5: Assembly of Dbfp9 and overlapping de novo spectra
- Figure S3: Alignment of C-terminus Dbfp9 fragments

Dbfp2 Assembly

- Table S6: Fragments of Dbfp2 identified from the whole TP extract using LC-MS/MS, and proposed assembly

1. Quagga mussel foot transcriptome library construction

RNA was extracted from three quagga mussels, to account for allelic variation in the Lake Ontario local population, each sequenced with its own unique barcodes. A sufficient sequencing depth of over 10 Giga-bases of data per sample was achieved; over 90% of bases identified with a Phred score higher than 30, indicating a high-quality raw data set. Following trimming and removal of low-quality reads, data from all three samples were pooled for *de novo* assembly using Trinity. A minimum 90% of reads from each sample were successfully mapped to the assembled library, suggesting successful high-quality assembly. The RNA-sequencing results and *de novo* assembly are summarized below in **Table S1**.

Table S1: Summary of RNA-sequencing statistics and assembly quality.

**QM Sample**	**Reads** (#)	**Trimmed reads** (#)	**Yield** (Mbases)	**% of Bases >Q30**	**Mean Quality** (Phred Score)	**Read Map Percent**
1	68,924,000	67,704,022	10,223	93%	35.15	92%
2	92,294,744	89,892,366	13,574	91%	34.64	90%
3	90,174,006	85,308,846	12,882	90%	34.27	92%

Mind Trinity assembled the long continuous regions of DNA (contigs) into 122,606 components (analogous to genes), containing 207,239 transcripts, analogous to protein variants or isoforms. For all transcripts, the median contig length was 386 bp, and the N30 and N50 contig lengths are 2396 bp and 1481 bp, respectively. The assembly of long continuous regions of DNA further suggests the assembly is of high quality. The distribution of contig lengths is shown below in Figure 3-1. The cDNA library was translated in all six reading frames to create the quagga mussel transcriptome protein library utilized to fingerprinting proteins sequences using LC-MS/MS.

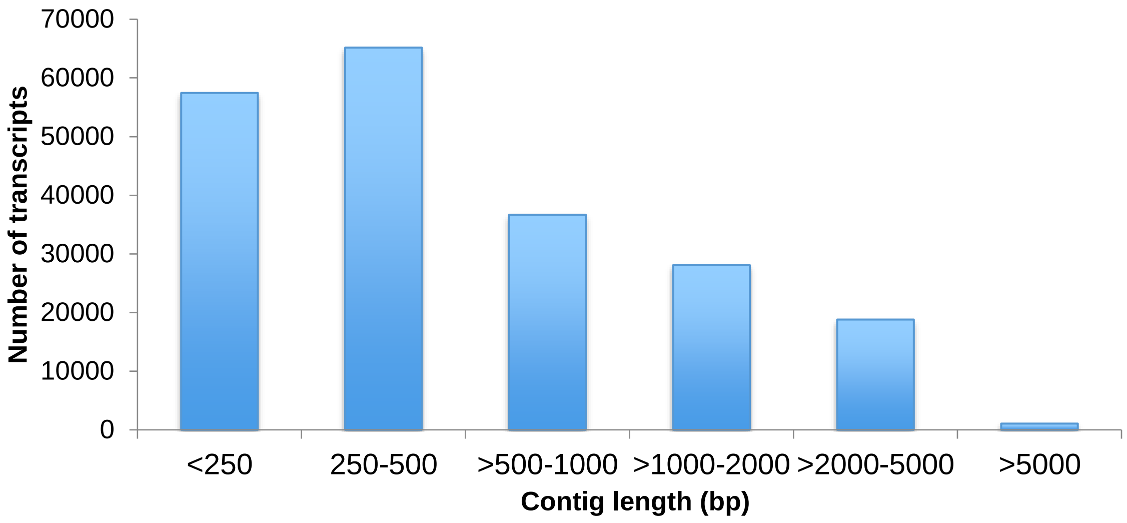

Figure S1: Distribution of transcript lengths in quagga mussel transcriptome library.

2. Quagga mussel gel bands removed for LC-MS/MS

Due to the extensive cross-linking in the mature quagga mussel byssus, mussels were induced to secrete fresh byssal material that was isolated and analyzed. Soluble byssal proteins separated by Tris-Bis SDS-PAGE (**Figure S2**). Four gel bands were clipped from each of the two lanes and pooled for LC-MS/MS analysis: ~6, ~7, ~14, and ~28 kDa, respectively.

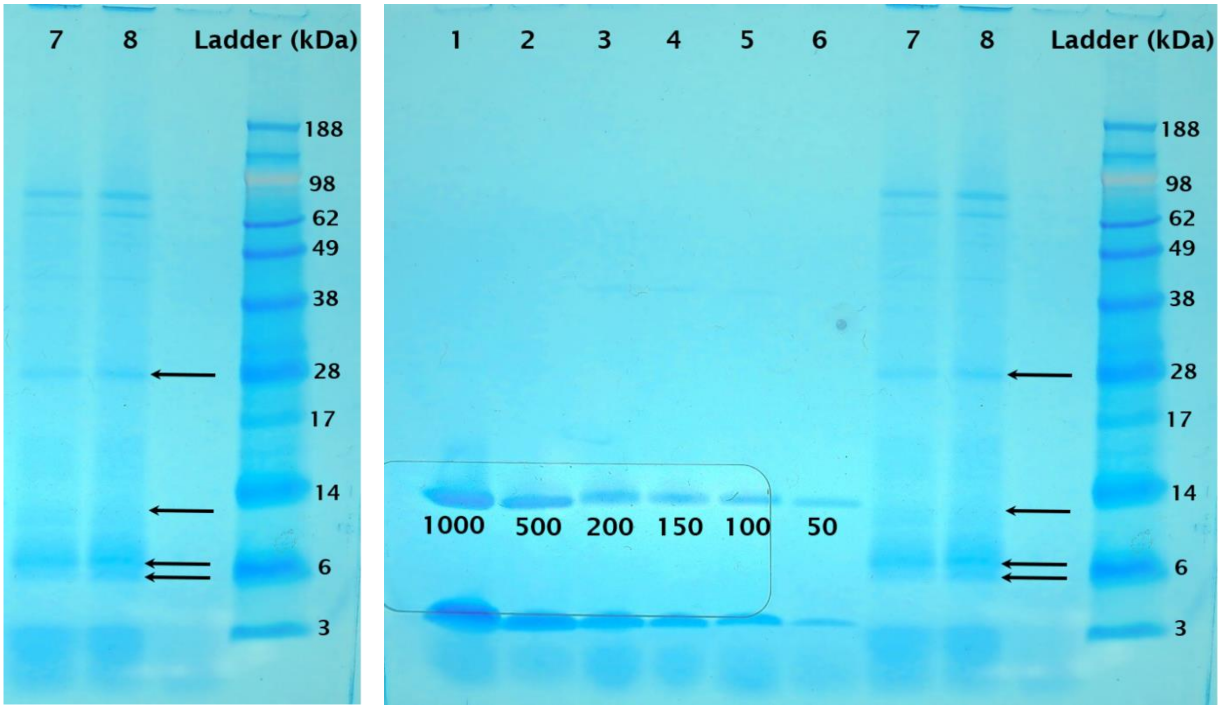

Figure S2: (left) SDS-PAGE gel visualized with SimplyBlue. Lanes 7 and 8 each contain 10TP equivalent of QM soluble protein (1.2µg). The ~6, ~7, ~14, and ~28 kDa clipped bands for LC-MS/MS are highlighted with arrows. (right) Uncropped gel, lanes 1-6 are irrelevant to this experiment.

3. Quagga mussel protein assembly using transcriptome library

Table S2: Additional large proteins with a high number of spectral counts identified by LC-MS/MS that did not meet acceptance criteria to be listed as a novel quagga mussel byssal protein.

[TABLE S2 ATTACHED AT END OF DOCUMENT]

Table S3: Byssal proteins identified by LC-MS/MS analysis using the foot transcriptome library. Signal peptides are underlined & matching peptide spectra are bolded.

[TABLE S3 ATTACHED AT END OF DOCUMENT]

Dbfp1 – Mining the transcriptome library for additional fragments

By searching the transcriptome library for known Dbfp1 sequences (DKYPGGG), multiple components and transcripts were observed that appear to be fragments of Dbfp1. This revealed one component containing multiple transcripts that are fragments of Dbfp1 named Dbfp1-f3. A component transcript of Dbfp1-f3 contained the start codon and signal peptide of the protein, which is predicted for secretion. After trimming the signal peptide, the N-terminus sequence is GVPEYTTKSPVYT, similar to the N-terminal sequence of Dbfp1 previously observed by Waite et al: GVPEYYN?[Q/G]PV[Y/V]Y. This component was not observed using LC-MS/MS, however it clearly is the beginning of Dbfp1, and as such was included here. Mining the transcriptome library, we also found the Dbfp1 C-terminus terminating at a stop codon named Dpfp1-f4. Although Dbfp1 was not fully assembled in the library, the essence of the protein has been determined. **Table S4** below depicts the various Dbfp1 fragments and how they could potentially be assembled.

Table S4: Dbfp1 fragments and proposed overlapping sequences.

Dbfp1 Fragment	Transcript sequence
**Dbfp1-start comp34314_c0_seq1-0R-f1**	MLIAVTFLLLAACCGTSLSGVPEYTTKSPVYTT<br>KLPEYPTKYPTYPT
**Dbfp1-start comp34314_c0_seq2-1R**	KLPEYPTQYPTYPDVTIGPVYPTQND<br>KYPGDGNDKYPGGGD
**Dbfp1-f2 comp9617_c0_seq2-2R**	KYPGDGNPKYPGGGNPKYPGGGNPKYPGGGNPKYPGGGN<br>AKYLGGGNDKYP… (likely many repeats)
**Dbfp1-end comp30958_c0_seq1-f4**	DKYPGGGSGYGDPDPYFPGGPWGK-stop

Dbfp9 – Manual assembly of using de novo only spectra

The Dbfp9 start (signal peptide and start codon) was observed with full coverage in the whole TP extract, 6 kDa, and 7 kDa bands, shown below with signal peptide removed. The largest C-terminus transcript with the second highest coverage, Dbfp9-fb is shown below. There are mismatched asparagine (N) and lysine (K) residues. However, since a single base substitution can result in asparagine being replaced by lysine and vice-versa, this could be explained by base substitution errors when creating the transcriptome cDNA library from the mussel mRNA. De novo-only spectra strongly suggest the alignment is correct, and the sequence actually contains either a lysine or asparagine residue. There are 8 high-quality (>80% ALC) de novo sequences (labeled DN1 through DN8) which span the conjunction between the Dpfp9 start and end transcripts, shown below in **Table S5**. Aspartic acid (D) and Asparagine (N) have a similar side-chain with a mass difference of only 0.98 Da, therefore a residue labelled N+0.98 is most likely an aspartic acid reside. Asparagines that were identified as N+0.98 are underlined as D to make the alignment clearer.

Table S5: Assembly of Dbfp9 and overlapping de novo spectra.

Fragment	Peptide sequence
**Dbfp9 start**	FVYGDYDDDYGYGGNYG
**Dbfp9-fb**	DDYGYGGKYGYPGNYGYP…
**DN1**	DYDDDYGYGGK
**DN2**	YGDRYDDDYGYGGK
**DN3**	FVYGDYDDDYGYGGK
**DN4**	GDYDDDYGYGGK
**DN5**	GDYDDDYGYGGK
**DN6**	YDDDYGYGGK
**DN7**	DDYGYGGK
**DN8**	DYGYGGK

This C-terminus component was used to assemble Dbfp9. Three transcripts from two other components were also aligned with Dbfp9-fb (Dbfp-fa, Dbfp9-fg, Dbfp9-fh).

Other additional interesting sequences with similarities to Dbfp9 includes a variant from the component containing the C-terminus of Dbfp9, containing the sequence NYGYPGYGG

repeated 5 times (highlighted in blue and green below). This variant was only fingerprinted by 1 spectra in the 7kDa band data, and was incomplete (no start or end codon), and was not included in the variant list. Another interesting sequence mined from the library that is similar to the Dbfp9 start codon sequence, contains the sequence shorter sequence GGNY repeated 9 times (highlighted in yellow and pink). No spectra were observed by LC-MS/MS confirming the existence of the sequence on the protein-level, so it was excluded from the analysis. However, RSEM analysis suggests these transcripts are expressed to a similar level as Dbfp16. Due to their similarity to Dbfp9 the sequences have been included below.

Dbfp9 transcript (comp42199_c1_seq5-1F):

NYGYPGYGGNYGYPGYGGNYGYPGYGGNYGYPGYGGNYGYPGYGGNYGYPG

Dbfp9 potential start (comp37514_c0_seq4-0F):

MCLLVAAAVLLAVANARSVPYYGDYGYGGNYGGNYGGNYGGNYGGNYGGNYGGNYGGNYGGNY

Many additional Dbfp9 C-terminus fragments were fingerprinted by LC-MS/MS spectra in both the whole TP and gel band data. Dbfp9-fa and Dbfp9-fb overlapped with the Dbfp9 transcript containing the start codon and could be successfully assembled. The additional Dbfp9 fragments did not overlap with the start codon. However, the sequences were all identified by multiple LC-MS/MS spectra and RSEM analysis suggests they are significantly expressed. Furthermore, the additional transcripts demonstrate sequence variation in the C-terminus of Dbfp9, thus we believe they warrant inclusion. Using Dbfp9-fb as a reference the fragments were aligned with Clustal Omega to assemble additional Dbfp9 variants to demonstrate polymorphism of Dbfp9, and the possible range in mass of the protein variants (**Figure S3)**. Using the proposed methods to assemble the additional variants, Dbfp9 could range from 6.6 – 9.0 kDa.

```
Dbfp9-fa:      YGYGGNYGGNYGGNYGRNYGWPGNYGYDGNNGYPGYYGGQGNYGGYDNYLGGWLGQLLGG     60
Dbfp9-fb:      ----------DDYGYGGKYGYPGNYGYPGNSGYPGNYGGYGNYGD--NDYGGWLGGLLGG     48
Dbfp9-fc:      -----------------------NYGYPGNSGYPGNYGGYGNYGD--NNYGGWLGGLLGG     35
Dbfp9-fd:      ------------------------YGGYGNSGYPGNYGGYGNYGD--NDYGGWLGGLLGG     34
Dbfp9-fe:      ------------------------YGG------YGNYGGYGNYGD--NDYGGWLGGLLGG     28
Dbfp9-ff:      --------------------------YGGNSGYPGNYGGY----D--NNYGGWLGGLLGG     28
Dbfp9-fg:      --------------------YPGNYGYSGNSGHPGNYGGKGNYGYPGGNYGK--------     32
Dbfp9-fh:      --------------------------YPGNSGYHGHYGGKGNYGYPGGNYGK--------     26
                                                 * ***           *

Dbfp9-fa:      GGKG-----KWDNYGYGK-      73
Dbfp9-fb:      GGRGNKWGGNYGNYGYGK-      66
Dbfp9-fc:      GGKGNKWGGNYGKYGYGK-      53
Dbfp9-fd:      GGRGNKWGGNYGNYGYGK-      52
Dbfp9-fe:      GGRGNKWGGNYGNYGYGK-      46
Dbfp9-ff:      GGRG-----KWGNYGYGKK      42
Dbfp9-fg:      G--------KSGDYRYGKY      43
Dbfp9-fh:      G--------KSGDYRYGKY      37
               *        :  .* ***
```

Figure S3: Alignment of C-terminus Dbfp9 fragments. Fragments β (b) through ε (e) are transcripts from the same library component. Fragment β (b) was the longest observed fragment with the highest coverage and was used as a base to align the other components, as shown in Appendix E. Fragment α (a), η (g) and θ (h) are from different library components.

Dbfp2 Assembly

Dbfp2 has a highly repetitive block structure. The Dbfp-f2 transcript contains an octapeptide sequence YPTYPEKK consecutively repeated five times, shown below in **Table S6**. *De novo* assembly during the transcriptome construction likely could not fully assemble the protein because the highly repetitive YPTYPEKK domain at the DNA level is beyond the resolution of the 150bp paired-end Illumina sequencing. Dbfp2-f1 contains the signal peptide and start codon and ends with a single YPTYPEKK sequence. Dbfp-f3 has the same motif of YPTYPEKK observed four times, highlighted in green and blue in **Table S6**. The Dbfp-f3 C-terminus contains a triple tandem repeat of the sequence YPDYPEKK, highlighted in pink and yellow. A consensus sequence combining both of these motifs is YP(T/D)Y(P/T)EKK, where the tyrosine, lysine, and glutamic acid residues are highly conserved. Tyrosine positioning throughout the central region Dpfb2 is highly conserved, with the pattern YxxYxxxxY, where x represents any amino acid.

Table S6: Fragments of Dbfp2 identified from the whole TP extract using LC-MS/MS, and proposed assembly.

Transcript	Peptide sequence
**Dbfp2-f1 comp9618_c0_seq1-2R**	MLSSVTLLFVACCGMALGQGNSWDSYRPYPVYTPKPSYPDYPEKPYPP KQT **Y**PT**Y**PEKK
**Dbfp2-f2 comp12693_c0_seq 3-1F**	**Y**PT**Y**PEKK**Y**PT**Y**PEKK**Y**PT**Y**PEKK**Y**PTYPEKK **Y**PT**Y**PEKK**Y**PT**Y**PEK
**Dbfp2-f3 comp12693_c0_seq 1-1F**	**Y**PT**Y**PEKK**Y**PT**Y**PEKK**Y**PT**Y**PEPT**Y**PT**Y**TEKK**Y**PA**Y**TPKT**Y**PT**Y**TEKK **Y**PD**Y**PEKK**Y**PD**Y**PEKK**Y**PD**Y**PEKK**Y**PQ**Y**K
**Dbfp2-f4 comp31091_c1_seq 1-0R**	T**Y**PEKK**Y**PS**Y**PEKK**Y**PA**Y**PPKNSYPGRYPWRR-stop

Note: Tandem repeats are highlighted in green/blue, and yellow/magenta respective. Tyrosine residues have been bolded to emphasize their highly conserved position. The underlined region of Dbfp2-f1 is the signal peptide predicted to be cleaved. Transcripts are staggered to demonstrate overlap of the transcripts used to assemble Dbfp2.

Table S2: Additional large proteins with a high number of spectral counts identified by LC-MS/MS that did not meet acceptance criteria to be listed as a novel quagga mussel byssal protein.

**Library transcript**	**MW**[a] (kDa)	**pI**[a] (pH)	**Protein Start Signal, End** (Yes, No)	**Score**[b] (-10LogP)	**Spectral Matches** (#)	**BLAST Match** (Protein, Species)	**BLAST E value**
comp56420_c14_seq2-1F	209.0	3.9	N/N/Y	235.2	54	Rho termination factor [Plasmodium falciparum IGH-CR14]	1e-15
comp48282_c0_seq2-2R	68.8	7.4	Y/Y/Y	173.39	36	Putative tyrosinase-like protein tyr-3 [Crassostrea gigas]	2e-52
comp53523_c0_seq1-1F + comp53523_c1_seq2-0R	130.3	6.2	Y/Y/Y	216.4	35	Predicted: kielin/chordin-like protein isoform X3 [Lingula anatina]	3e-64
comp53833_c0_seq1-1R	190.6	4.4	Y/Y/N	177.0	33	Predicted: mucin-17-like [Crassostrea gigas]	0
comp53519_c2_seq1-2R	124.6	5.1	N/N/N	182.79	31	IgGFc-binding protein [Crassostrea gigas]	4e-102
comp56544_c0_seq1-2R	149.2	6.2	Y/Y/N	185.88	29	Predicted: kielin/chordin-like protein isoform X2 [Lingula anatina]	1e-76
comp56544_c3_seq1-2R	231.3	6.8	N/N/Y	205.21	27	Predicted: kielin/chordin-like protein isoform X2 [Lingula anatina]	1e-150
comp54843_c0_seq1-0R	214.9	26	Y/Y/N	160.75	26	Predicted: SCO-spondin-like [Xenopus (Silurana) tropicalis]	0
comp52859_c0_seq2-1R	114.6	4.3	Y/N/Y	161.37	20	Predicted: mucin-5AC-like [Crassostrea gigas]	2e-116
comp55322_c0_seq2-0F	209.3	5.0	N/N/Y	158.52	21	Predicted: SCO-spondin-like [Octopus bimaculoides]	0
comp53849_c0_seq1-1R	211.7	5.3	Y/Y/N	161.37	20	Galaxin precursor - reef coral [Galaxea fascicularis]	1e-49
comp53998_c1_seq1-0R	189.9	5.3	Y/Y/Y	150.29	18	Predicted: matrilin-4 isoform X9 [Poecilia formosa]	6e-55
comp47861_c0_seq1-0F	169.4	6.1	N/N/Y	157.44	17	Predicted: kielin/chordin-like protein isoform X2 [Lingula anatina]	3e-100
comp58164_c0_seq4-1R	118.6	7.5	N/N/Y	130.94	16	Galaxin [Exaiptasia pallida]	2e-66
comp54122_c0_seq1-2F	111.5	4.4	N/N/Y	130.47	13	hypothetical protein DQ04_00171070 [Trypanosoma grayi]	4.2
comp56286 c1 seq1-1R	51.0	4.7	Y/Y/Y	121.77	10	Predicted: EGF-like domain-containing protein 1 [Aplysia californica]	2e-20
comp53132_c0_seq2-2R + comp53132_c1_seq1-2F	51.1	6.7	Y/Y/Y	109.53	10	Putative tyrosinase-like protein tyr-3 [Crassostrea gigas]	9e-74
comp37709_c0_seq1-0R	79.5	8.3	Y/Y/Y	96.02	6	Galaxin precursor - reef coral [Galaxea fascicularis]	4e-60
comp50133_c1_seq4-2F	56.2	7.0	N/N/N	115.43	6	Predicted: kielin/chordin-like protein [Tursiops truncatus]	4e-45

[a] Sequence properties were calculated after removing the predicted signal peptide sequence
[b] PEAKS scoring method: a -10LogP score cut-off of 20 is equivalent to P-value of 0.01

Table S3: Byssal proteins identified by LC-MS/MS analysis using the foot transcriptome library. Signal peptides are underlined & matching peptide spectra are bolded.

Protein name	Library transcript	Protein sequence	MW, pI[a]	Score[c] (-10LogP)	LC-MS/MS Spectral Matches (#) TP	6kDa	7kDa	14kDa
**Dbfp1-s1**	comp34314_c0_seq1-0R	MLIAVTFLLLAACCGTSLSGVPEYTTKSPVYTTKLPEYPTKYPTYPT	3.2 kDa pI 8.9	-	0	0	0	0
**Dbfp1-s2**	comp34314_c0_seq2-1R	KLPEYPTQYPTYPDVTIGPVYPTQNDKYPGDGNDKYPGGGD	4.5 kDa pI 4.0	-	0	0	0	0
**Dbfp1-r1**	comp39272_c0_seq1-0R	WNDKYPGDGKDKYPGDGDDK**YLGGGNDKYLGGVFDK**YFGGGN	4.6 kDa pI 4.4	75.07	3	1	1	1
**Dbfp1-r2**	comp9617_c0_seq2-2R	KYPGDGNPKYPGGGNPKYPGGGNPKYPGGGNPKYPGGGNAKYLGGGNDKYP	5.1 kDa pI 10	27.12	1	0	0	0
**Dbfp1-end**	comp30958_c0_seq1-2F	DKYPGGGSGYGDPDPYFPGGPWGK	2.5 kDa pI 4.3	-	0	0	0	0
**Dbfp2-start**	comp9618_c0_seq1-2R	MLSSVTLLFVACCGMA**LGQGNSWDSYRPYPVYTPKPSYPDYPEKPYPPKQTYPTYPEKK**	5.1 kDa pI 9.2	90.13	9	1	3	2
**Dbfp2-mid**	comp12693_c0_seq3-1F	**YPTYPEKKYPTYPEKK**YPTYPEKKYPTYPEKKYPTYPEKKYPTYPEK	5.8 kDa pI 9.7	55.96	4	1	2	1
**Dbfp2-mid**	comp12693_c0_seq1-1F	**YPTYPEKKYPTYPEKK**YPTYPEP**TYPTYTEKKYPAYTPK**TYPTYTEK**KYPDYPEKK**YPDYPEKKYPDYPEKKYPQYK	9.7 kDa pI 9.4	84.08	10	1	2	1
**Dbfp2-end**	comp31091_c1_seq1-0R	TYPEKK**YPSYPEKKYPAYPPKNSYPGRYPW**RR	4.0 kDa pI 10.2	62.53	4	3	3	1
**Dbfp4**	comp37554_c0_seq1-1F	MFGLVAVSVFLFCHSSA**FSNTWQNR**IK**QRPTPVVPF**KLE**WYLGKWFTQSRQEPCSWKGSADFENMELNFVLDPKKNILYDHSIWKK**NNR**CVFVTFDIIPSPK**TPGTFLIQDPLGDIQSGEYVILAIDPCK**FVVEWGCTKPSPIGQRCDDPWVSVHTRERPSPKVLAEVDLALMR**TVGVRLAELPR**LSHANTPCCLGEGKLIQHDFL**	21.8 kDa pI 8.1	149.48	20	0	0	0

Protein name	Library transcript	Protein sequence	MW, pI[a]	Score[c] (-10LogP)	LC-MS/MS Spectral Matches (#)			
					TP	6kDa	7kDa	14kDa
**Dbfp5α**	comp47359_c0_seq3-2R	MFSAVTLVLLVSCCGTALSQRNSYGNYRPVKPPGQPINQYNQYSNPYRPQYNQNWNPYRPEQAPR**YPQQSYPAYPPK**QPYPAYPTKQPYPTDPPKQPYPANPSKPSYPANPPYDPCDEVYCRPIYCPNGQYKPTGECCPQCQPGTYLPKPWSWRGQGNVVGEQEK**FVGEGNVVGDQTYDVGGQGNVVGGQR**NVVDGKGNVVGEQRNNVGG	21.3 kDa pI 8.7	85.71	3	1	0	0
**Dbfp5β**	comp43939_c0_seq2-0F	MFSAVTLMLFVSCCGTALS**ELDPYWNSYLPK**NPLSTASIK**NNQYWN**SYRTEQSPQQYVPVYAPYDPCYEVR**CPPIICR**YGHMRPQGKCCPHCIPDLYFSRFPSEKWGWQGNFDGEQINDVGGQGNSVGLQGNDVR**GKGNVVGWQGNVVDGQGNVVGEQ**RNGVDGQGN	15.6 kDa pI 5.7	84.67	4	0	0	0
**Dbfp6**	comp31337_c0_seq1-0F	MFSAASFLLLVMFCGTVTS**QFYWGYLPQRLYPRDPCDDVDCRTPHCPNGGYIPIGQCCPK**CKPAASWALEVTLHVFSGRPDPEYVIPR**DTSAYDAILKAIGDTSTPLGER**LGYNGFTVIQTHGDSEVSHWTVGWCTRPKVELR**LLAAVSAMTPIGDQHPLQKEVIDTVKQSIMLCKV (154)**	17.6 kDa pI 6.6	110.57	9	1	1	1
**Dbfp7α**	comp52765_c1_seq1-1F	MFFAVTLVLLVSCSGTPLGK**WDPYGSSYGNSYGRPYGKAFNPYNQYGNSYPQNNQKWNSYWPNYKQPWNSYGPQQYPSYPQ**SGSYYPGSWGWPGNNVGSQGN**AVDGLWNVVGWQGNDVDGLGNNVGKQWNDVDGVGNYVGKQWNNVD**	14.8 kDa pI 6.6	134.09	16	10	10	9
**Dbfp7β**	comp52765_c1_seq5-1F	MFFSAVVLVLLVSCSGTPLGKWDPYGNSYGNPY**AFNPYNQYGNSYPQNNQKWNSYWPNYKQPWNSYGPQQYPSYPQ**SGSYYPGSWGWPGNNVGSQGNAVDGLWNVVGWQGNDVDGLGNNVGK**QWNDVDGVGNYVGKQWNNVD**	14.2 kDa pI 4.3	103.12	10	9	9	9
**Dbfp7γ**	comp52765_c1_seq9-1F	MFFSAVVLVLLVSCSG**TPLGKWDPYGLSYGNPYGKALNPYNQYGNQYWPQYKQPWNSYGPQQYPSYPQKGTYWPGGWGFPGNNVGSQGN**AVDGLWNVVGWQGNDVDGLGNNVGK**QWNDVDGVGNYVGKQWNNVD**	13.2 kDa pI 4.7	147.16	25	20	19	16
**Dbfp7δ**	comp52765_c1_seq8-1F	MLSAVILVLLVSCS**GTPLGQWDPYGKAVNPYNKYGNQYWPQYKQPWNSYGPQQYPSYPQKGTYWPGGWGFPGNNVGSQGN**AVDGLWNVVGWQGNDVDGLGNNVGK**QWNDVDGVGNYVGKQWNNVD**	12.3 kDa pI 4.7	131.81	22	18	19	16

Protein name	Library transcript	Protein sequence	MW, pI[a]	Score[c] (-10LogP)	LC-MS/MS Spectral Matches (#)			
					TP	6kDa	7kDa	14kDa
**Dbfp7ε**	comp52765_c1_seq13-1F	MFFSAVVLVLLVSCSG**TPLGKWDPYGNSYGNPYGKALNPYNQYGNQ**YWPQYN**QPWNSYGPQQYPSYPQKGSYLPGGWGWPGNNVGKQWNDVDGVGNYVGKQWNNVD**	10.3 kDa pI 8.7	129.34	18	8	10	9
**Dbfp7ζ**	comp52765_c1_seq3-0F	LVLLVSCS**GTPLYGNSYGNPYGKALNPYNQYGNQYWPQYKQPWNSYGPQQYPSYPQKGSYLPGGWGWPGNNVGKQWNDVDGVGNYVGKQWNNVD**	9.7 kDa pI 9.1	152.89	27	16	17	13
**Dbfp7 fragment**	comp30312_c0_seq1-1R	VGSQGN**AVSGIGNVVGSQR**NNVGGQGNNVGWQGNDVSGVGNFVGSQDNDVSGQGNNVGWQR**NGVSGLGNFVGK**QWNSVN	7.1 kDa pI 6.5	56.25	2	0	0	0
**Dbfp8α**	comp44862_c1_seq2-2F	MKLALLAVIAFVAPSCYEATYPVPNQGRCLKDGQYFASGH**FVDPTNR**CTSCECFPGGNYQCRRDACPELSCPVNQR**FYPHDACCQR**CHGVIHSPGSASSVSSSDHDTSGTSRHTSKSSKSSRGTSKNSKSSKKSSKSKSSRKGSKGSRKGSRKGSRKGYRKGSKKYGKKGSGSSS **(156)**	16.7 kDa pI 10.7	50.61	2	0	0	0
**Dbfp8β**	comp44862_c1_seq3-2F	MKLALLAVIAFVAPSCYEATYPVPNQGRCLKDGQYFASGH**FVDPTNR**CTSCECFPGGNYQCRR**DACPALSCPVNQRFYPHDACCQR**CHGVIHSPGSASSVSSSDHDTSGTSRHTSKSSKSSRGTSKNSKSSKKSSKSKSSRKGSKGSRKGSRKGSRKGYKKYGKKGSGSSS **(152)**	16.3 kDa pI 10.6	68.3	3	0	0	0
**Dbfp9α**	comp58518_c0_seq1-1R + comp31072_c0_seq1-2F	MNTKQLMCLLVAAALLLASAPAANA**RFVYGDYDDD**YGYGGN**YGGNYGGNYGRNYGWPGNYGY**DGNNGYPGYYGGQGN**YGGYDNYLGGWLGQLLGGGGKGKWDNYGYG**K	9.0 kDa pI 4.3	83.56	6	3	1	0
**Dbfp9β**	comp42199_c1_seq4-1F + comp31072_c0_seq1-2F	MNTKQLMCLLVAAALLLASAPAANA**RFVYGDYDDDYGYGGKYG**YPGNYGYPGNSGYP**GNYGGYGNYGDNDYGGWLGGLLGGGGR**GNKWGGNYGNYGYGK	7.9 kDa pI 4.6	106.28	9	9	7	1
**Dbfp9γ**	comp42199_c1_seq2-1F + comp31072_c0_seq1-2F	MNTKQLMCLLVAAALLLASAPAANA**RFVYGDYDDDYGYGGKYG**YPGNYGYPGN**SGYPGNYGGYGNYGDNNYGGWLGGLLGGGGKGNKWGGNYGK**YGYGK	7.8 kDa pI 8.6	93.15	4	8	10	1
**Dbfp9δ**	comp42199_c1_seq9-0F + comp31072_c0_seq1-2F	MNTKQLMCLLVAAALLLASAPAANA**RFVYGDYDDDYGYGGKYG**YPGNYGGYGNSGYP**GNYGGYGNYGDNDYGGWLGGLLGGGGR**GNKWGGNYGNYGYGK	7.8 kDa pI 4.6	106.28	9	6	7	1

Protein name	Library transcript	Protein sequence	MW, pI[a]	Score[c] (-10LogP)	LC-MS/MS Spectral Matches (#)			
					TP	6kDa	7kDa	14kDa
**Dbfp9ε**	comp42199_c1_seq8-0F + comp31072_c0_seq1-2F	MNTKQLMCLLVAAALLLASAPAANA**RFVYGDYDDDYGYGGKYG**YPGNYGGYGNYGGY**GNYGDNDYGGWLGGLLGGGGR**GNKWGGNYGNYGYGK	7.2 kDa pI 4.6	106.28	9	6	6	1
**Dbfp9ζ**	comp42199_c1_seq3-0F + comp31072_c0_seq1-2F	MNTKQLMCLLVAAALLLASAPAANA**RFVYGDYDDDYGYGGKYG**YPGNYGYGGN**SGYPGNYGGYDNNYGGWLGGLLGGGGR**GK**WGNYGYG**KK	7.1 kDa pI 8.6	139.25	11	5	11	3
**Dbfp9η**	comp35873_c0_seq1-2F + comp31072_c0_seq1-2F	MNTKQLMCLLVAAALLLASAPAANA**RFVYGDYDDDYGYG**GKYGYPGNYGYPGNSGYHGHYGGK**GNYGYPGGNYGKGKSGDYRYGKY**	6.7 kDa pI 9.0	38.57	1	2	4	1
**Dbfp9θ**	comp35873_c0_seq2-1F + comp31072_c0_seq1-2F	MNTKQLMCLLVAAALLLASAPAANA**RFVYGDYDDDYGYG**GKYGYPGNYGYSGNSGHPGNYGGK**GNYGYPGGNYGKGKSGDYRYGK**Y	6.6 kDa pI 9.0	38.57	1	2	4	1
**Dbfp10α**	comp35857_c0_seq1-0R	MQSAVTLLLLVSCCGMALGQWDDY**DDWDWPTGYPSYPPKQSYPPYPPYDPCKNVNCIQVVCPYGEYTPPGK**CCPVCIDWGWPWGPYGSSGSDDYDDDDDYWPYNWGK	10.3 kDa pI 3.5	101.96	4	0	0	0
**Dbfp10β**	comp35857_c0_seq3-0R	MLSAVTLLLLVSCCGMALGQRDYWPPWDPCPC**CAVLCAEIFCPYGQYIPKGQCCPVCK**DWIWPWRPYWSSGLSGSDDNYWPNNWGK	7.9 kDa pI 4.7	63.86	4	2	3	2
**Dbfp10γ**	comp40957_c0_seq1-2F	MFSAASFLLLVMFCGTVTSQPYWPYPTSRPYPIK**NCLAVDCPPVYCPYGQYIPK**GECCPRCKKGYPYPDPYFPGGK	6.6 kDa pI 8.6	70.56	3	1	2	2
**Dbfp10δ**	comp35857_c0_seq2-0R	MLSAVTLLLLVSCCGMALGGDYWPPWDPCDDLICMPIVCPYGWYTPPGECCPVCIDWGWPWEPYGSSGSDDYDDDDDYWPYNWGK	7.7 kDa pI 3.1	0	0	0	0	0
**Dbfp11α**	compxv_c4_seq2-0R	MCSATPFLLLVTFCGTVNS**IWYPYRTATTPPRPCYGVKCPPIYCPYGQVTPPGKCCPQCKPAPGGDIHVPCKTDK**GCKDVVCDDPREKVECHEDVDSPTGSACHCHIPGECVNDWDCFKECGRDATCDDGACHGSSCDHT	13.1 kDa pI 5.5	102.58	7	0	0	0
**Dbfp11β**	comp49590_c4_seq3-0R	MCSATPFLLLVTFCGTVSS**LWYPNRPCYGKVCPAIYCLY**GEVTPPGECCPQCKPDPSSNIHKPCYK**DKDCDCVVCDNPWEEVECHDAPSSPTGRE**CHCHRPGECDNDYDCYFECGPGATCDDGACHGPWCDHT	12.7 kDa pI 4.4	105.65	4	0	0	0

Protein name	Library transcript	Protein sequence	MW, pI[a]	Score[c] (-10LogP)	LC-MS/MS Spectral Matches (#)			
					TP	6kDa	7kDa	14kDa
**Dbfp11γ**	comp49590_c4_seq4-0R	MCSATPFLLLVTFCGAVSS**LWYPDRPCYGKVCPAIYCLYGQVTPPGK**CCPQCKPDPGSNVHVPCKK**DKDCAYVVCENPGEKVECHDAPSSYPPRR**ECHCHIPEECEKDFDCVDECGPGATCDDGACHGNDCDHT	12.7 kDa pI 4.7	137.49	12	0	0	0
**Dbfp11δ**	comp49590_c4_seq6-0R	QTMCSATPFLLLVTFCGTVSS**LWYPNRPCYGKVCPAIYCLYGQVTPPGK**CCPQCKPDPGSNVHVPCKKDKDCAYVVCENPGEEVECHDDVDSPTGRACHCHIQGECVNDWDCFKECGRDATCDDGACHGSSCDHT	12.5 kDa pI 4.9	102.78	8	0	0	0
**Dbfp11ε**	comp49590_c4_seq1-0R	MCSATPFLLLVTFCGAVSS**LWYPDRPCYGKVCPAIYCLYGQVTPPGKCCPQCKPAPGGDIHVPCKTDK**GCKDVVCDDPREKVECHEDVDSPTGSACHCHIPGECVNDWDCFKECGRDATCDDGACHGSSCDHT	12.4 kDa pI 4.9	117.81	10	0	0	0
**Dbfp12α**	comp45612_c1_seq1-0F	MALSTWSLFMIVAATMYTGSCQQCPVGTPLKECEGFTQGHDRPWCPVGYYCRDLWMNNPGICCRKVCWDGPPSTDKYNRVIDCSRGRK**GLCPEATECVR**YGRYGARSFCCNIRVTIG	10.9 kDa pI 8.3	53.6	1	0	0	1
**Dbfp12β**	comp45612_c1_seq6-0F	MALSTWSLFLIVAATMYTGSCQECPVGSILKGCEFIQGHEGPWCPVGYYCRDLWMNNPGICCRKVCWDGPPSTDKYNRVIDCSRGRKG**LCPEATECVR**YGRYGARSFCCNIRVTIG	10.7 kDa pI 8.1	53.6	1	0	0	1
**Dbfp12γ**	comp45612_c1_seq2-0F	MALSTWSLFLIVAATMYTGSCQQCPVGTPLKECEGFTQGHDRPWCPVGYYCRDLMMNNLGICCR**NVCWDGPPITDNYGRA**IDCSRGR**TGLCPGATECVR**YGRYGAR**SFCCNIR**VTIG	10.7 kDa pI 8.0	67.76	3	0	0	3
**Dbfp12δ**	comp45612_c1_seq5-0F	MALSTWSLFLIVAATMYTGSCQECPVGSILKGCEFIQGHEGPWCPVGYYCRDLMMNNLGICCR**NVCWDGPPITDNYGR**AIDCSRGR**TGLCPGATECVR**YGRYGAR**SFCCNIR**VTIG	10.5 kDa pI 7.8	119	3	0	0	3
**Dbfp13α**	comp49016_c0_seq15-1R	MKGVFLLLAIVCIMVEAGGRRNQRPMYRRRLPPPTTKKPPPRPTPAPTQAPR**GGPYEHEQLTQNIEK**QLKEMNTTLDAIYTLTNEMFTIRNKCLDAYRPSG	9.6 kDa pI 10.8	52.4	2	0	0	0
**Dbfp13β**	comp49016_c0_seq2-1R	MVFQSMKGVFLLLAIVCIMVEAGGRRNRYQPRNQRPKPTTKPPPRPTPAPTQAPR**GGPYEHEQLTQNIEK**QLKEMNSTLDAIFTLSNEMFKLKNKCLDAYGPSG	9.2 kDa pI 10.6	52.4	2	0	0	0

Protein name	Library transcript	Protein sequence	MW, pI[a]	Score[c] (-10LogP)	LC-MS/MS Spectral Matches (#) TP	6kDa	7kDa	14kDa
**Dbfp13γ**	comp49016_c0_seq12-2R	MVFQSMKGVFLLLAIVCIMVEAGGRRRLPPPTTKKPPPRPTPAPTQAPR**GGPYEHEQLTQNIEK**QLKEMNSTLDAIFTLSNEMFKLKNKCLDAYGPSG	8.3 kDa pI 10.2	52.4	2	0	0	0
**Dbfp13δ**	comp49016_c0_seq11-2R	MVFLSIKGVFLLLAIAFIVVEAQWRGRPPPTRPTPPPR**GGSNGHEQLTQNIEK**QLKEMNTTLDAIYTLTNEMFT**IRNKCLD**AYRPSG	7.4 kDa pI 9.9	42.59	1	0	0	0
**Dbfp13ε**	comp9630_c0_seq1-0R	MVFLSIQGVFLLLIIACIVMDAGAPRPPPTRPTPPQR**GGSYGHEQLTQNIEK**QLKEMNK**ILDAIYTLTNEMFK**IRNKCLD	7.1 kDa pI 10.0	66.13	3	0	0	0
**Dbfp14α**	comp35860_c0_seq2-2R	MGPNKLFVTVLIICLMGAMVAGQDDR**AAGSDWPGCWCR**WRRCR**SNECDLGWCPWDWGRR**RLCCR**FFSSHCGWR**NEP	6.6 kDa pI 8.2	98.22	5	0	0	0
**Dbfp14β**	comp35860_c0_seq6-2R	MGPNKLFVTVLIICLMGAMVAGQDDR**AAGPDWPMCYCR**WIRCR**WNECDLGDR**GSRFCGMGRRLCCRLFSRHCGWRN	6.5 kDa pI 8.6	59.92	2	0	0	0
**Dbfp14γ**	comp35860_c0_seq1-2R	MGPNKLFVTVLILCLMGAM**GGSDDPPAAPGR**CYCR**RVCRPNECYVGR**CPRRR**SWSWCCHR**SHPDCEGK	5.6 kDa pI 8.6	89.84	6	6	14	7
**Dbfp14δ**	comp35860_c0_seq4-2R	MGPNKLFVTVLILCLVGAMGGGGDDAQPAAPGSCHCRWR**CNPNECYVR**RCRGMRSWCCHRSHPDCEGK	5.4 kDa pI 8.2	27.86	1	0	1	1
**Dbfp15α**	comp37514_c0_seq1-2F	MCLLVAAAVLLAIAPIANAKYGSSSDSSDSDGYNGKRGGYGR**RGGLPWPR**YGRGK**GYGGWGDNYGAVPT**YGK	5.5 kDa pI 9.9	69.24	0	0	2	1
**Dbfp15β**	comp37514_c0_seq2-2F	MCLAAAVLLAIAPIANAKYGSSSDSSDSDGYNGRRGGYGGRPRYGRGK**GYGGWGDNYGAVPTY**GK	5.0 kDa pI 9.9	48.77	1	0	1	0
**Dbfp15γ**	comp37514_c0_seq3-0F	MCLLVAAAVLLAIAPIANATYGSSSDSSDSDGYSGRRGGHGGRRGGYGGRGGLLWPRYGRVGRYGGLGGSYGAYGK	5.8 kDa pI 10.4	-	0	0	0	0
**Dbfp16**	comp13545_c0_seq2-1F	MFSAVVTVLIICLMGVMGGGDREPAGPDWWCDWRWQCSR**NECVVFEDFGGKFCCLWTSPLCWSKD**R	5.3 kDa pI 4.4	68.66	3	1	1	0
**Dbfp17**	comp12176_c0_seq2-2R	MTSVRILVVLMVVCILAGSVVQQAEAQCHMTLK**GCANNECFTGTVGK**RKKCCPK**KNNACPQGLPSV**	4.3 kDa pI 9.2	47.92	2	0	0	0
